# Associations of thiazide use with skin cancers: a systematic review and meta-analysis

**DOI:** 10.1186/s12916-022-02419-9

**Published:** 2022-07-07

**Authors:** Shih-Chieh Shao, Chien-Cheng Lai, Yi-Hung Chen, Edward Chia-Cheng Lai, Ming-Jui Hung, Ching-Chi Chi

**Affiliations:** 1 Department of Pharmacy, Chang Gung Memorial Hospital, Keelung, Taiwan; 2 School of Pharmacy, Institute of Clinical Pharmacy and Pharmaceutical Sciences, College of Medicine, National Cheng Kung University, Tainan, Taiwan; 3 College of Medicine, Chang Gung University, Taoyuan, Taiwan; 4 Division of Cardiology, Department of Internal Medicine, Chang Gung Memorial Hospital, Keelung, Taiwan; 5 Department of Dermatology, Chang Gung Memorial Hospital, Linkou, Taoyuan, Taiwan

**Keywords:** Thiazides, Hydrochlorothiazide, Bendroflumethiazide, Indapamide, Non-melanoma skin cancer, Melanoma, Systematic review, Meta-analysis

## Abstract

**Background:**

Previous findings on the associations of thiazide use with skin cancers were conflicting. This study aimed to examine the associations of individual thiazide use with skin cancer risk, differentiated by subtypes of skin cancers, geographic regions, and cumulative doses of individual thiazides.

**Methods:**

We searched PubMed, Embase, and Cochrane Central Register of Controlled Trials for relevant studies on January 5, 2022, scanned the references of included studies, and consulted experts. We included case-control and cohort studies or randomized trials reporting the associations of individual thiazide or thiazide-like diuretics use with skin cancers. Non-melanoma skin cancer (NMSC) and melanoma were analysed separately. A random-effects model meta-analysis was conducted for pooled odds ratio (OR) and hazard ratio (HR) for skin cancers related to individual thiazide use.

**Results:**

We included 15, 5, and 5 case-control or cohort studies reporting the risk for skin cancers associated with hydrochlorothiazide, bendroflumethiazide, and indapamide use, respectively, with 17,848,313 participants. The meta-analysis showed associations of hydrochlorothiazide use with increased risk of NMSC (OR 1.16, 95% CI 1.08–1.24; HR 1.26, 95% CI 1.04–1.54), squamous cell carcinoma (SCC) (OR 1.32, 95% CI 1.06–1.65; HR 1.61, 95% CI 0.97–2.67), and melanoma (OR 1.11, 95% CI 1.02–1.20; HR 1.03, 95% CI 0.93–1.14). The increased risks for SCC were associated with high cumulative doses of hydrochlorothiazide (OR 2.56, 95% CI 1.43–4.57; HR 1.20, 95% CI 1.00–1.45). Hydrochlorothiazide use was associated with different subtypes of melanoma including superficial spreading (OR 1.18, 95% CI 1.05–1.33), nodular (OR 1.23, 95% CI 1.08–1.39), and lentigo maligna melanoma (OR 1.33, 95% CI 1.08–1.65). Various cumulative doses of hydrochlorothiazide were associated with increased odds for melanoma. However, the associations of hydrochlorothiazide use with increased risk of NMSC and melanoma only appeared in non-Asian countries. No meaningful increase in the risk for skin cancers was associated with bendroflumethiazide and indapamide.

**Conclusions:**

Hydrochlorothiazide is associated with an increased risk for NMSC (especially SCC) and melanoma in non-Asian countries, whereas bendroflumethiazide and indapamide are not associated with a meaningful risk for skin cancers. Healthcare professionals and patients should be informed of the different risk profiles of skin cancers associated with different thiazides, cumulative doses, and regions.

**Trial registration:**

PROSPERO CRD42021234317.

**Supplementary Information:**

The online version contains supplementary material available at 10.1186/s12916-022-02419-9.

## Background

According to the Global Burden of Disease Study, the incidences of basal cell carcinomas (BCC), squamous cell carcinomas (SCC), and melanoma have increased by 77%, 309%, and 161%, respectively, from 1990 to 2017 [1]. Identification and avoidance of modifiable risk factors may mitigate this increasing trend. One risk factor is the interaction of sunlight with medications, leading to photosensitivity responses in susceptible patients, potentially increasing the risk of skin cancer [2, 3].

Among first-line antihypertensives [4], thiazide diuretics have photosensitizing properties with a biologically plausible causal association with skin cancers [5]. Previous Danish studies have suggested an increased risk for non-melanoma skin cancers (NMSC) and some melanoma subtypes associated with hydrochlorothiazide [6, 7]. Several systematic reviews have indicated overall thiazide uses increased the risk of skin cancers [5, 8, 9]. However, the risk profiles may vary between different individual thiazides. For example, indapamide and bendroflumethiazide did not pose additional risk of skin cancers, based on a recent study by Schneider et al. [10]. Furthermore, studies from Asian countries have shown findings inconsistent with those from non-Asian countries which indicated ethnic differences between Caucasians and Asians with regard to adverse effects of medications. For example, hydrochlorothiazide appears safe for use in Taiwanese and Korean populations [11, 12], while Australian and Icelandic studies have found significant associations of thiazide use with skin cancers [13, 14]. Thus, this study aimed to examine the associations of individual thiazide use with skin cancer risk, differentiated by subtypes of skin cancers, geographic regions, and cumulative doses of individual thiazides.

## Methods

We followed the Preferred Reporting Items for Systematic Reviews and Meta-analyses (PRISMA) and Meta-analysis of Observational Studies in Epidemiology (MOOSE) guidelines [15, 16]. Two authors (CCL and YHC) independently performed study selection, data extraction, and risk of bias (RoB) assessments. Two senior authors (SCS and CCC) helped resolve disagreements. The protocol was registered with PROSPERO (CRD42021234317).

### Literature search

We searched PubMed, Embase, and Cochrane Central Register of Controlled Trials for relevant studies published from inception to January 5, 2022. We also screened the bibliographies of included studies and consulted experts for relevant unpublished studies. Additional file 1: Table S1 presents the search strategy.

### Study selection

We developed the participants (P), interventions (I) or exposures (E), comparators (C), and outcomes (O) framework for study selection. For RCTs or cohort studies, the PICO included P: patients without skin cancers; I: any individual thiazide use; C: no thiazide use or controls; O: skin cancers. For case-control studies, the PECO included P: patients with skin cancers; E: any individual thiazide use; C: no thiazide use or controls; O: skin cancers. The skin cancers were defined as NMSC [17, 18] (including BCC, SCC, and Merkel cell carcinoma (MCC)) and melanoma.

The exclusion criteria were as follows: (1) other types of publications (case reports, narrative reviews, systematic reviews, editorials, guidelines, and viewpoint papers); (2) studies from spontaneous adverse drug reaction reports databases; (3) studies on transplant populations receiving immunosuppressants with increased risk for skin cancers [19]; (4) duplicate studies from an overlapping population with a smaller sample size and ancient time span [20–22]; and (5) studies with major methodological weaknesses in the study design, as determined by two review authors (ECCL and CCC, who have doctorate training in pharmacoepidemiology, clinical epidemiology, and evidence-based medicine) and one experienced expert in this field (AP; see the “Acknowledgements” section).

### Expert opinions

We examined the author lists in the included studies, and contacted the expert (AP) who was most frequently listed in this field for unpublished or missing literature and for discussion of the severe methodological weaknesses in the included studies.

### Data extraction and risk of bias assessment

The following data were extracted: study design, first author, publication year, country, mean age, sex, photosensitive co-medications, comorbidities, and risk estimates on the association of thiazide use with skin cancers. For studies lacking relevant outcome data, we contacted the authors for clarification.

The RoB in the included observational studies and RCTs was assessed using the Newcastle-Ottawa Scale (NOS) and Cochrane RoB 2.0 tool, respectively [23–25]. We considered studies with a score of 9 stars to be at low RoB, studies that scored 7 or 8 stars at moderate RoB, and those that scored 6 stars or less at high RoB. Specifically, the NOS provides the same reliability as other RoB assessment tools [26], and it is commonly used to judge the methodological quality of included observational studies in previous systematic review and meta-analysis [27–29].

### Statistical analysis

If a study provided multiple risk estimates, those with the most fully adjusted confounders were adopted. The meta-analysis was conducted using Review Manager Version 5.4 (Cochrane Collaboration, 2020) to investigate the associations of different thiazides with skin cancers, and the risk for NMSC (BCC, SCC, MCC, and unspecified NMSC) and melanoma was calculated. The pooled risk ratio (RR) with 95% confidence interval (CI) was calculated for RCTs and pooled odds ratio (OR) with 95% CI for case-control studies. Incidence rate ratio was considered hazard ratio (HR), and pooled HR with 95% CI were calculated for cohort studies [30]. The *I*^2^ statistic was used to quantify statistical heterogeneity across the included studies, whereby substantial heterogeneity was considered when the *I*^2^ statistic exceeded 50% [31]. Anticipating clinical heterogeneity, we performed a random-effects model meta-analysis. Inverse-variance-weighted method was used for pooling results to estimate the risk estimates of skin cancer associated with individual thiazide use. Subgroup analyses covering melanoma subtypes (superficial spreading, nodular, and lentigo maligna melanoma), geographic regions (Asian and non-Asian), and cumulative thiazide doses (high, medium, and low) were performed to examine the individual risk profiles. For studies not reporting the skin cancer risk from different cumulative doses of individual thiazide use, we analysed the different cumulative treatment durations, where available. To determine the robustness of the results of our main analyses, we conducted a sensitivity analysis by including duplicate studies from the overlapping population and another sensitivity analysis by including only studies with low risk of bias. Publication bias would be evaluated by assessing funnel plot asymmetry if there were ≥ 10 studies included in a meta-analysis on a study outcome [32]. However, publication bias evaluation was not performed because there were < 10 studies for all outcomes on individual thiazide uses.

## Results

### Characteristics of included studies

**Fig. 1:**
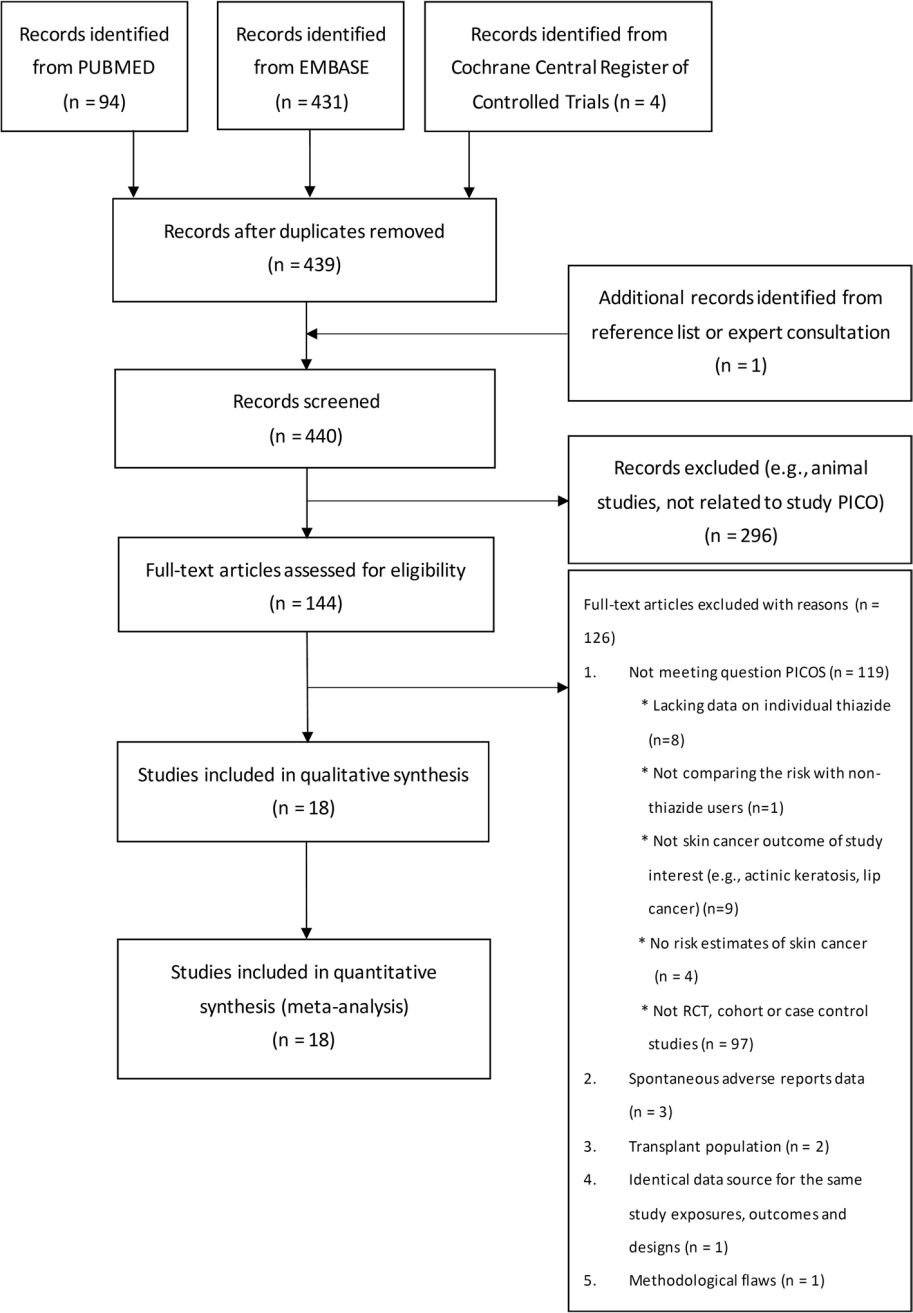
PRISMA study flowchart

Figure 1 shows the PRISMA study flowchart. The studies with overlapping populations and selections of studies in the main analyses are presented in Additional file 1: Table S2. In addition, one cohort study with immortal time bias highlighted in a commentary written by Pottegård et al was excluded [33–35]. Briefly, because hydrochlorothiazide was less likely to be prescribed as the drug of choice for antihypertensive treatment, following up patients from the first date of hydrochlorothiazide may lead to a spurious protective effect against skin cancers because participants were free from the outcome of skin cancer (i.e., immortal time) until they received hydrochlorothiazide. Finally, 13 case-control studies [6, 7, 11, 13, 14, 36–43] and 5 cohort studies [10, 12, 44–46] with a total of 17,848,313 participants, with 6,790,008 exposed to thiazides, were included, and most of the studies analysed hydrochlorothiazide-containing drugs. The summary for the inclusion of thiazides is presented in Additional file 1: Table S3. No relevant RCTs were found. We found 15 [6, 7, 10–14, 36–40, 43, 45, 46], 5 [7, 10, 41, 42, 44], and 5 observational studies [7, 10, 14, 37, 42] on skin cancer risk associated with hydrochlorothiazide, bendroflumethiazide, and indapamide use, respectively. These studies were from 17 countries, and the study characteristics are listed in Table 1 and Additional file 1: Table S4. The cumulative treatment duration cut-off points among the included studies were too heterogeneous to perform the meta-analysis, so we only reported the qualitative results of the relationship between cumulative duration of individual thiazide use and skin cancer risk (Additional file 1: Table S5).

**Table 1:** Characteristics of the included studies

First author, year (country)	Data sources	Study design	Case/exposed group (***n***)	Control/non-exposed group (***n***)	NOS score		
					S	C	E/O^a^
Tiba, 2022 (Brazil) [38]	Brazilian Association of Dermatology	Case control	NMSC (31)	Unaffected population (58)	2	2	1
Habel, 2021 (USA) [36]	Kaiser Permanente Northern California	Case control	Melanoma (MM: 9176; SSM: 2241; NM: 477; LMM: 377)	Unaffected population (MM: 264,781; SSM: 63,396; NM: 14,322; LM: 12,352)	4	2	2
Adalsteinsson, 2021 (Iceland) [13]	National Register of Iceland	Case control	BCC (4700); SCC (1013)	Unaffected population (BCC:47,292; SCC:10,367)	4	1	3
León-Muñoz, 2021 (Spain) [37]	Information System for Research in Primary Care (SIDIAP)	Case control	KC (75,096); melanoma (8235)	Unaffected population (KC: 739,004); melanoma (79,843)	4	2	3
	Base de Datos para la Investigación Farmacoepidemiológica en Atencion Primaria (BIFAP)		BCC (26,200); SCC (4863); melanoma (4661)	Unaffected population (BCC: 262,000; SCC: 48,630; melanoma: 46,610)			
Daniels, 2020 (Australia) [14]	New South Wales Cancer Registry, Repatriation Pharmaceutical Benefits Scheme	Case control	Melanoma (MM: 659; SSM: 199; NM: 137; LMM: 134)	Unaffected population (MM:12,446; SSM:4197; NM:3050; LM:3044)	4	2	3
Morales, 2020 (UK) [40]	The Health Improvement Network	Case control	BCC (89,088); SCC (7560); melanoma (11,185)	Unaffected population (BCC: 1,781,712; SCC:151,194; melanoma: 223,700)	3	2	3
Yeon, 2020 (Korea) [43]	National Health Insurance claim data	Case control	NMSC (4098)	Unaffected population (6467)	3	1	3
Pedersen, 2019 (Denmark) [39]	Multi-registers and registration system ^b^	Case control	MCC (97)	Unaffected population (1857)	4	2	3
Pottegård, 2019 (Taiwan) [11]	Taiwan’s National Health Insurance Research Database (NHIRD)	Case control	NMSC (23,703); melanoma (5192)	Unaffected population (NMSC: 237,030; melanoma: 51,920)	3	2	3
Pottegård, 2018 (Denmark) [6]	NA (letter)	Case control	Melanoma (MM: 19,273; SSM: 13,781; NM: 1695; LMM: 500)	Cancer-free population (MM: 192,730; SSM: 137,810; NM: 16,950; LM: 5000)	4	2	1
Pedersen, 2018 (Denmark) [7]	Multi-registers and registration system^b^	Case control	BCC (71,553); SCC (8629)	Unaffected population (BCC: 1,430,883; SCC: 172,462)	4	2	3
de Vries, 2012 (Several countries ^c^) [ 41]	Multi-centre records from 8 countries	Case control	BCC (94); SCC (99); melanoma (33)	Unrelated to skin cancer (136)	3	2	1
Jensen, 2008 (Denmark) [42]	Danish Cancer Registry, Civil Registration System	Case control	Melanoma (1010)	Unaffected population (4040)	4	1	3
de Haan-Du J, 2021 (Netherlands) [46]	Netherlands Cancer Registry, and the Dutch Personal Records Database	Cohort	Hydrochlorothiazide (11,165)	Other antihypertensive medicine users (59,329)	4	2	1
Eworuke, 2021 (USA) [45]	US Food and Drug Administration’s Sentinel System	Cohort	Hydrochlorothiazide (5,211,321)	Other antihypertensive medicine users (ACEi, 5,211,321)	4	2	2
Schneider, 2021 (UK) [10]	UK-based Clinical Practice Research Datalink (CPRD)	Cohort	Hydrochlorothiazide, bendroflumethiazide, indapamide (271,154)	Other antihypertensive medicine users (CCB, 275,263)	4	2	2
Lee, 2020 (Korea) [12]	Observational Health Data Sciences (3 medical centres)^d^	Cohort	Hydrochlorothiazide (149,599)	Other antihypertensive medicine users (517,749)	4	2	1
Kaae, 2010 (Denmark) [44]	Danish Cancer Register, Civil Registration System	Cohort	Bendroflumethiazide (NA)	Never use of the bendroflumethiazide (NA)	2	2	2

*ACEi*, angiotensin-converting enzyme inhibitors; *BCC*, basal cell carcinoma; *C*, comparability; *CCB*, calcium channel blocker; *E*, exposure; *KC*, keratinocyte carcinoma; *LMM*, lentigo maligna melanoma; *MCC*, Merkel cell carcinoma; *MM*, malignant melanoma; *NA*, not available; *NM*, nodular melanoma; *NMSC*, nonmelanoma skin cancer; *NOS*, Newcastle–Ottawa Scale score; *S*, selection; *SCC*, squamous cell carcinoma; *SSM*, superficial spreading melanoma

^a^For case-control studies, this domain was exposure (E); for cohort studies, this domain was outcome (O)

^b^Danish Cancer Registry, National Prescription Registry, National Patient Registry, Danish Education Registers and Danish Civil Registration System

^c^Finland, Germany, Greece, Italy, Malta, Poland, Scotland, and Spain

^d^Seoul National University Hospital, Seoul National University Bundang Hospital, Asan Medical Center

### Risk of bias of included studies

The RoB in the included studies is summarized in Table 1 with detailed assessment presented in Additional file 1: Tables S6 and S7. With regard to the included case-control studies, most (4/13) did not report non-response rate for the exposed group while they did not employ nationwide data. We rated 4 case-control studies with overall low RoB (9 stars on the NOS), 7 case-control studies with overall moderate RoB (7 to 8 stars on the NOS) and 2 case-control studies with overall high RoB (≤ 6 stars on the NOS). With regard to included cohort studies, all (5/5) did not include adequate length of follow up (e.g., over 10 years) [47]. We judged 4 cohort studies with overall moderate RoB (7 to 8 stars on the NOS), and 1 cohort study with overall high RoB (≤ 6 stars on the NOS).

### Association between hydrochlorothiazide use and skin cancers

**Fig. 2:**
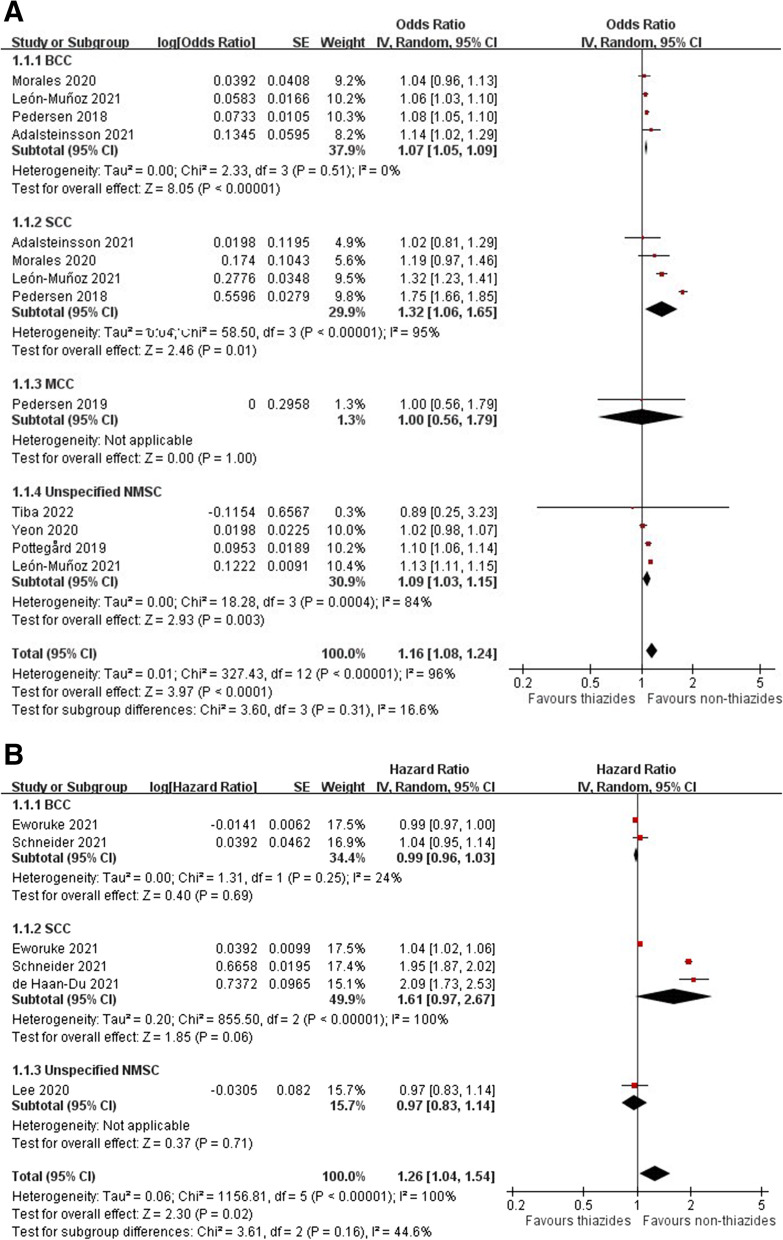
Forest plot on the association of hydrochlorothiazide use with nonmelanoma skin cancer: **A** case-control studies and **B** cohort studies

Eight case-control studies [7, 11, 13, 37–40, 43] and four cohort studies [10, 12, 45, 46] provided risk estimates for hydrochlorothiazide-associated NMSC. The meta-analysis demonstrated increased odds for NMSC among patients receiving hydrochlorothiazide in case-control studies (pooled OR 1.16, 95% CI 1.08–1.24, *I*^2^ = 96%) and cohort studies (pooled HR 1.26, 95% CI 1.04–1.54, *I*^2^ = 100%) (Fig. 2A, B). Among NMSC, an increased risk for hydrochlorothiazide-associated SCC (pooled OR 1.32, 95% CI 1.06–1.65, *I*^2^ = 95%; pooled HR 1.61, 95% CI 0.97–2.67, *I*^2^ = 100%) was found. However, no increased risk for hydrochlorothiazide-associated BCC (pooled OR 1.07, 95% CI 1.05–1.09, I^2^ = 0%; pooled HR 0.99, 95% CI 0.96–1.03, *I*^2^ = 24%) and MCC (OR 1.00, 95% CI 0.56–1.79) was found. The dose-response analyses (Additional file 1: Figures S1–S5) showed 2.56-fold (95% CI 1.43–4.57, *I*^2^ = 84%) and 1.20-fold (95% CI 1.00–0.45) increased risks for SCC associated with high cumulative doses of hydrochlorothiazide in case-control and cohort studies, respectively. The 1.23-fold (95% CI 1.07–1.41, I^2^ = 69%) increased risk for BCC associated with high cumulative doses of hydrochlorothiazide was only found in case-control studies, while there was no dose–response for BCC in cohort studies. We also found 3.30-fold (95% CI 1.31–8.31) increased risk for MCC associated with high cumulative doses of hydrochlorothiazide in one case-control study. However, one cohort study found no dose-response for unspecified NMSC (Additional file 1: Figure S6). We found 3 studies (2 case-control and 1 cohort studies) and 9 studies (6 case-control and 3 cohort studies) on the association of hydrochlorothiazide with NMSC from Asian and non-Asian regions, respectively. The meta-analysis revealed an increased risk for hydrochlorothiazide-associated NMSC in non-Asian countries (pooled OR 1.18, 95% CI 1.08–1.29, *I*^2^ = 97%; HR 1.33, 95% CI 1.07–1.65, *I*^2^ = 100%; Additional file 1: Figures S7A and S8A), contradicting the results from Asian studies (pooled OR 1.06, 95% CI 0.98–1.14, *I*^2^ = 85%; HR 0.97, 95% CI 0.83–1.14; Additional file 1: Figures S7B and S8B).

**Fig. 3:**
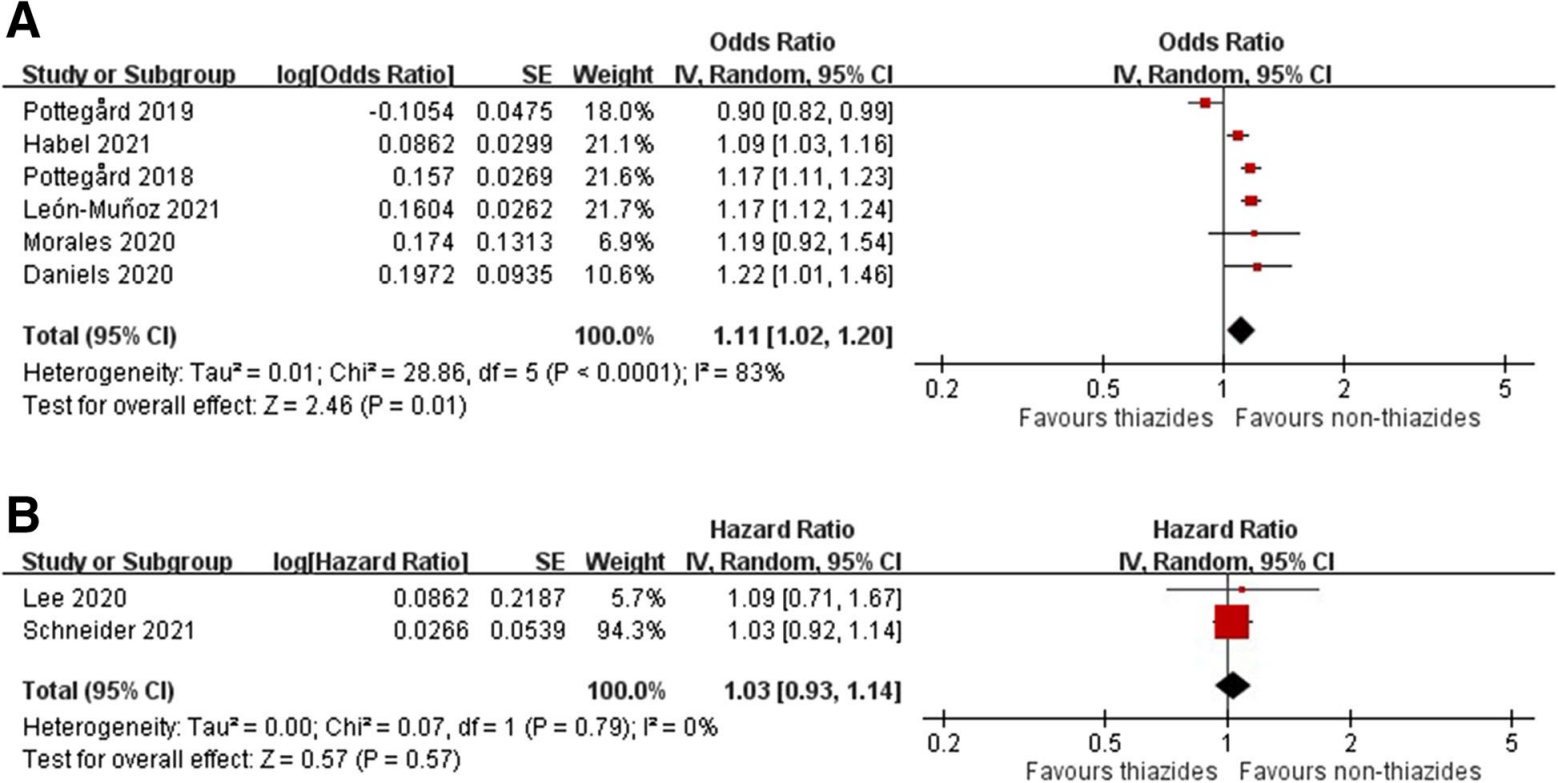
Forest plot on the association of hydrochlorothiazide use with melanoma: **A** case-control studies and **B** cohort studies

Six case-control studies [6, 11, 14, 36, 37, 40] and two cohort studies [10, 12]examined the association of hydrochlorothiazide use with melanoma. The meta-analysis revealed increased odds for melanoma among patients who received hydrochlorothiazide (pooled OR 1.11, 95% CI 1.02–1.20, *I*^2^ = 83%) in case-control studies, whereas no significant differences were observed in the cohort studies (HR 1.03, 95% CI 0.93–1.14, *I*^2^ = 0%) (Fig. 3A, B). Increased odds for melanoma in case-control studies were associated with high dose (pooled OR 1.15, 95% CI 1.07–1.24, *I*^2^ = 0%), medium (pooled OR 1.18, 95% CI 1.10–1.25, *I*^2^ = 3%), and low (pooled OR 1.09, 95% CI 1.01–1.19, *I*^2^ = 79%) cumulative doses (Additional file 1: Figure S9), while there was no dose-response for melanoma in one cohort study (Additional file 1: Figure S10).

We found two studies (one case-control study [11] and one cohort study [12]) and six studies (five case-control studies [6, 14, 36, 37, 40] and one cohort study [10]) on the association of hydrochlorothiazide with melanoma from Asian and non-Asian regions, respectively. One Taiwanese study with 317,845 subjects revealed reduced OR for hydrochlorothiazide-associated melanoma (OR 0.90; 95% CI 0.82–0.99), contradicting the results of five other non-Asian studies (pooled OR 1.14; 95% CI 1.10–1.19; *I*^2^ = 0%; test for subgroup differences: *P* < 0.00001; Additional file 1: Figure S11), whereas no risk for melanoma was observed both from Asian and non-Asian cohort studies (Additional file 1: Figure S12).

The subgroup analysis of the case-control studies revealed significant associations of hydrochlorothiazide with superficial spreading (pooled OR 1.18, 95% CI 1.05–1.33, *I*^2^ = 54%), nodular (pooled OR 1.23, 95% CI 1.08–1.39, *I*^2^ = 0%), and lentigo maligna melanomas (pooled OR 1.33, 95% CI 1.08–1.65, *I*^2^ = 37%). These were all non-Asian studies (Additional file 1: Figure S13).

### Association between bendroflumethiazide use and skin cancers

**Fig. 4:**
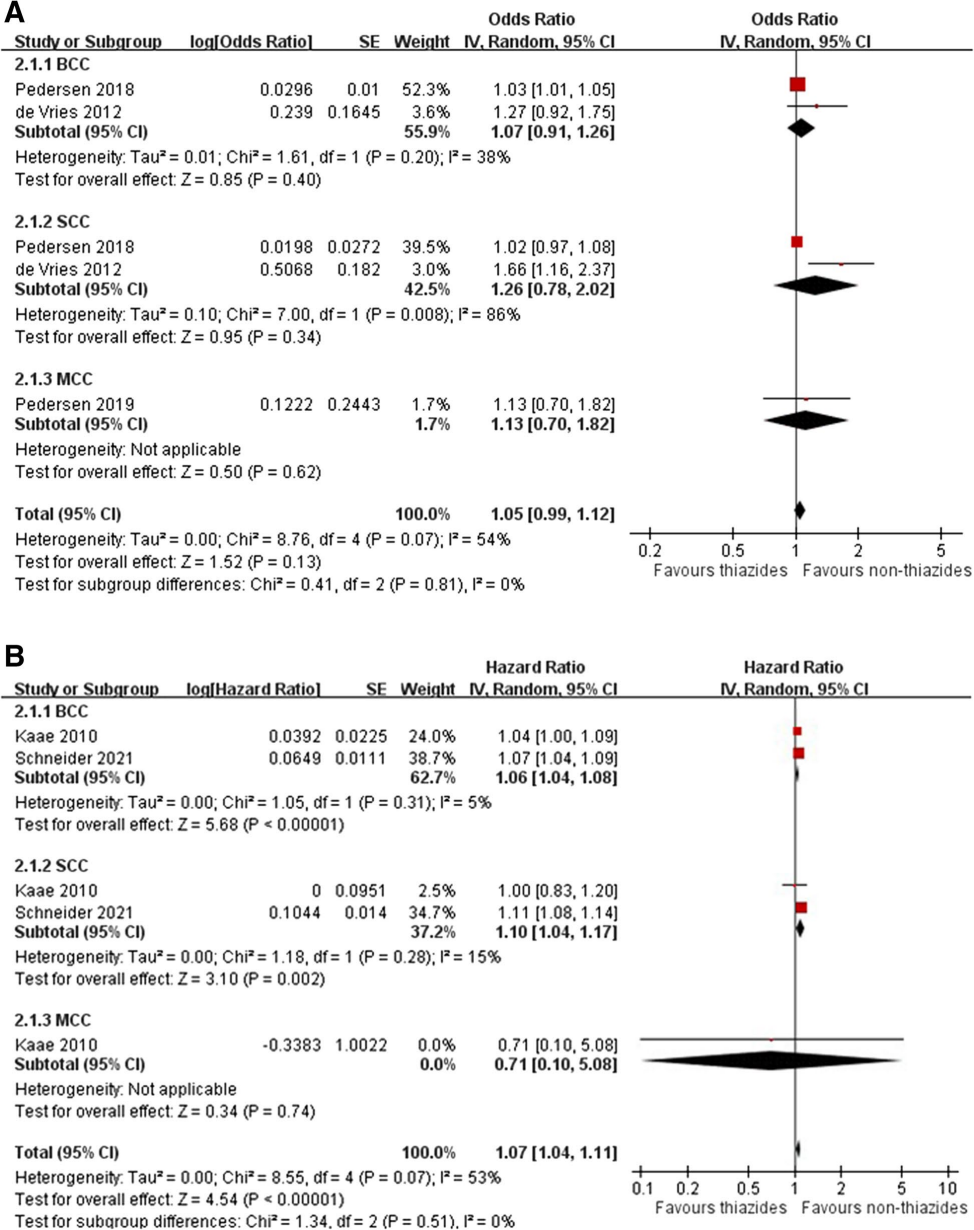
Forest plot on the association of bendroflumethiazide use with nonmelanoma skin cancer: **A** case-control studies and **B** cohort studies

Three case-control studies [7, 39, 41] and two cohort studies [10, 44] provided risk estimates for bendroflumethiazide-associated NMSC. The meta-analysis showed no increased odds for NMSC among patients receiving bendroflumethiazide (pooled OR 1.05; 95% CI 0.99–1.12; *I*^2^ = 54%) in case-control studies, whereas a significantly increased risk was observed in cohort studies (pooled HR 1.07, 95% CI 1.04–1.11, *I*^2^ = 53%) (Fig. 4A, B). No significantly increased risk for bendroflumethiazide-associated SCC (pooled OR 1.26, 95% CI 0.78–2.02, *I*^2^ = 86%; pooled HR 1.10, 95% CI 1.04–1.17, *I*^2^ = 15%) , BCC (pooled OR 1.07, 95% CI 0.91–1.26, *I*^2^ = 38%; pooled HR 1.06, 95% CI 1.04–1.08, *I*^2^ = 5%) and MCC (OR 1.13, 95% CI 0.70–1.82; HR 0.71, 95% CI 0.10–5.08) was found. The dose-response analyses on case-control studies found no significant or clinically meaningful increase in the risk for SCC, BCC, and MCC associated with higher cumulative doses of bendroflumethiazide (Additional file 1: Figures S14-S16).

**Fig. 5:**
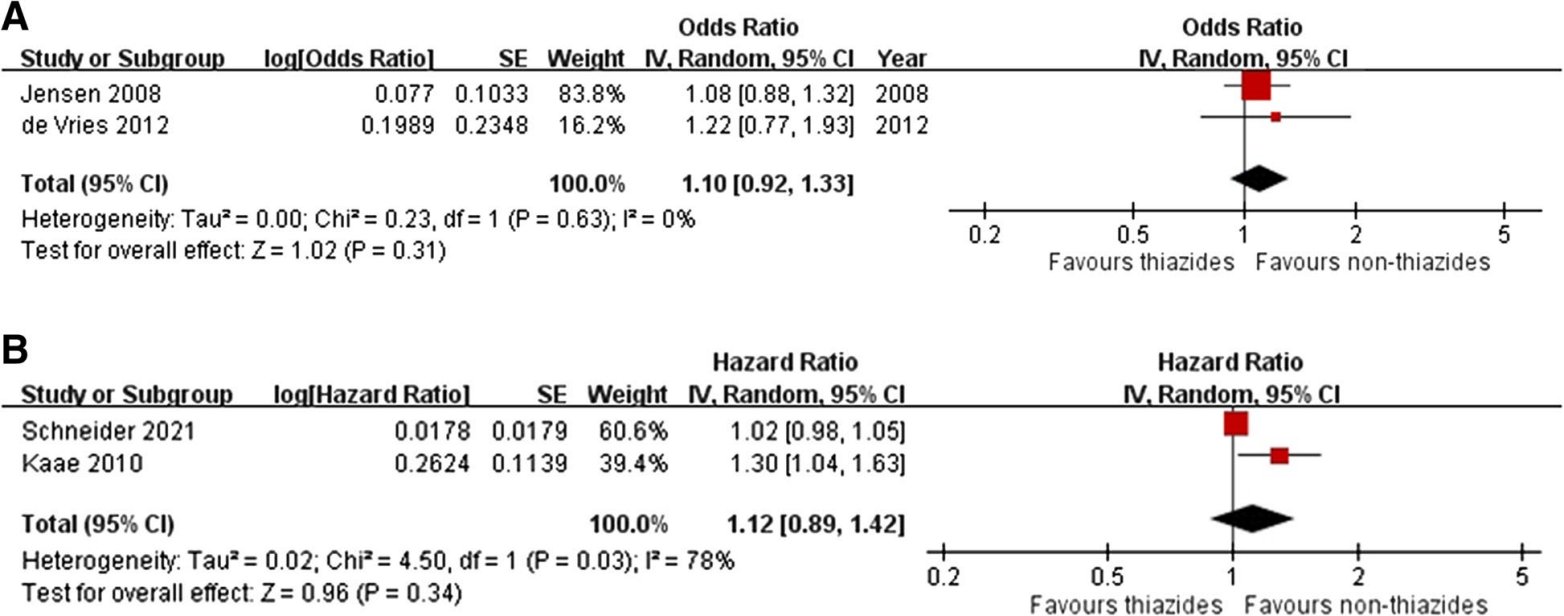
Forest plot on the association of bendroflumethiazide use with melanoma: **A** case-control studies and **B** cohort studies

Two case-control [41, 42] and two cohort studies [10, 44] provided data on the association of bendroflumethiazide use with melanoma. The meta-analysis indicated no increased risk for melanoma among patients receiving bendroflumethiazide in case-control (OR 1.10, 95% CI 0.92–1.33, *I*^2^=0%) and cohort (pooled HR 1.12, 95% CI 0.89–1.42, *I*^2^ = 78%) studies (Fig. 5A, B). The dose-response analyses on case-control studies found no significant or clinically meaningful increase in the risk for melanoma associated with higher cumulative doses of indapamide (Additional file 1: Figures S19). None of the included studies differentiated risks associated with bendroflumethiazide by melanoma subtypes, cumulative doses, or geographic regions.

### Association between indapamide use and skin cancers

**Fig. 6:**
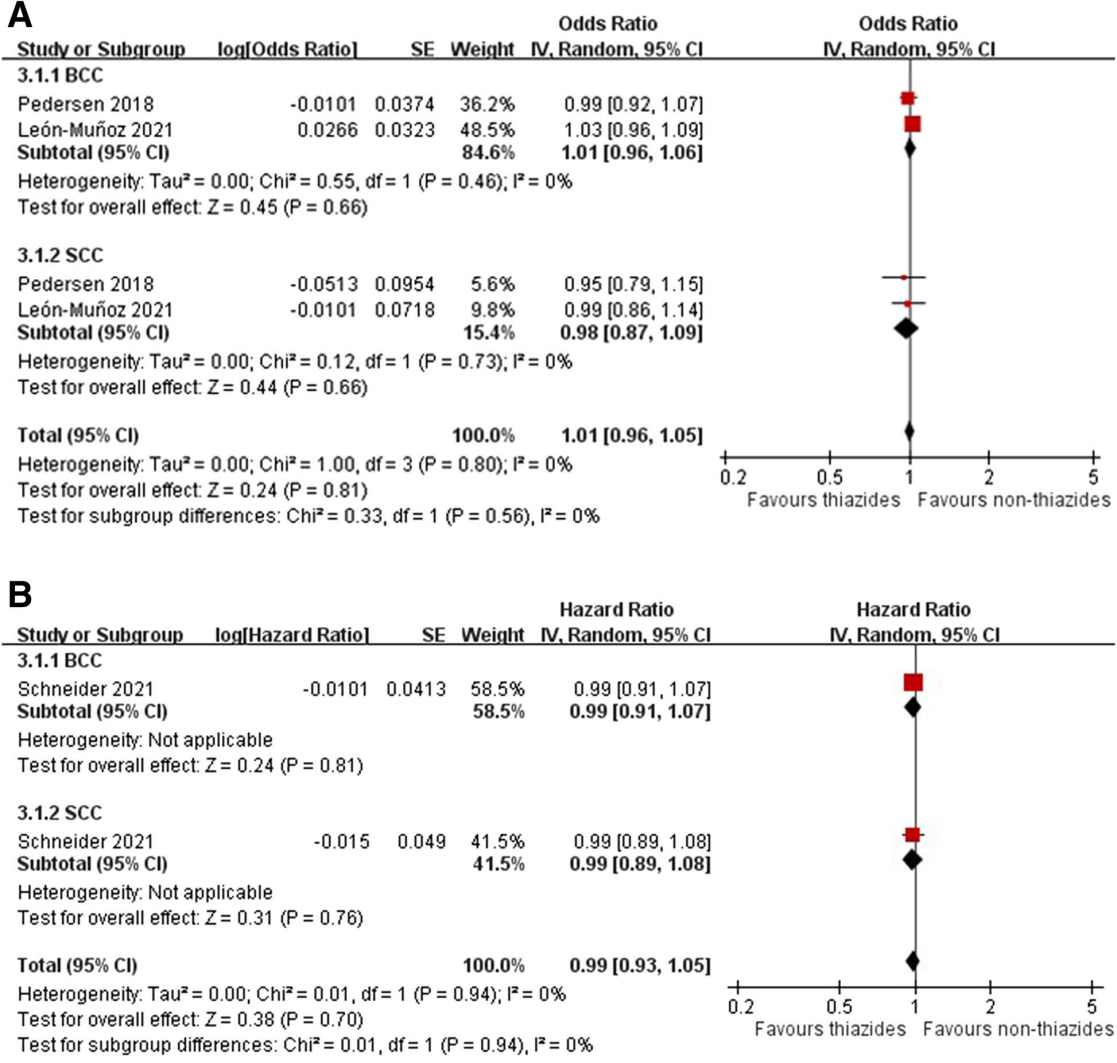
Forest plot on the association of indapamide use with nonmelanoma skin cancer: **A** case-control studies and **B** cohort studies

Two case-control studies [7, 37] and one cohort study [10] provided risk estimates for indapamide-associated NMSC. Two case–control studies showed no increased OR for NMSC among patients receiving indapamide (pooled OR 1.01, 95% CI 0.96–1.05, *I*^2^ = 0%), as did another cohort study from the UK (HR 0.99, 95% CI 0.93–1.05) (Fig. 6A, B). The risk for SCC associated with indapamide use (pooled OR 0.98, 95% CI 0.87–1.09, *I*^2^ = 0%; HR 0.99, 95% CI 0.89–1.08) was similar to that for BCC (pooled OR 1.01, 95% CI 0.96–1.06, *I*^2^ = 0%; HR 0.99, 95% CI 0.91–1.07). The dose-response analyses found no significant risk for SCC and BCC associated with higher cumulative doses of indapamide use (Additional file 1: Figures S17-S18).

**Fig. 7:**
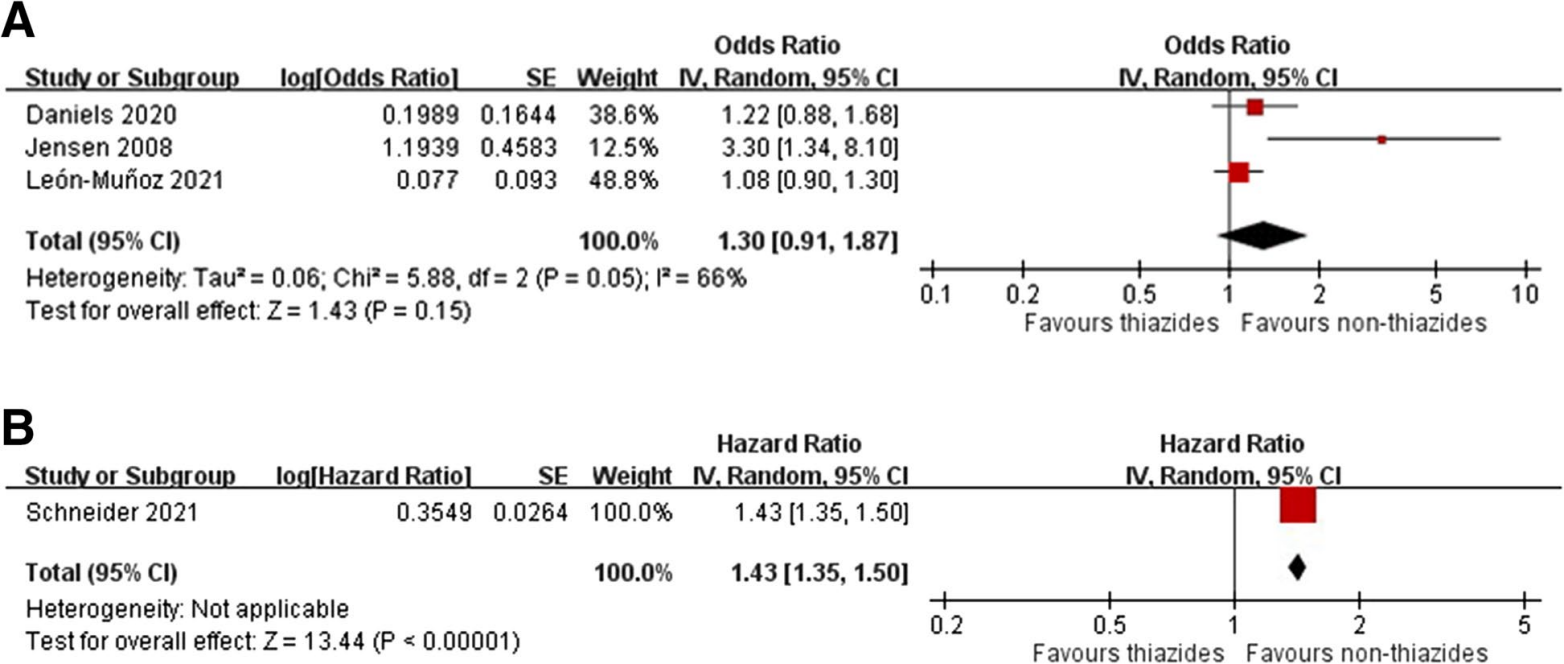
Forest plot on the association of indapamide use with melanoma: **A** case-control studies and **B** cohort studies

Three case-control studies [14, 37, 42] and one cohort study [10] provided data on the association of indapamide use with melanoma. The meta-analysis indicated no significant risk for melanoma among patients receiving indapamide in case-control studies (pooled OR 1.30, 95% CI 0.91–1.87, *I*^2^ = 66%), whereas one UK cohort study indicated a 43% risk increase (HR 1.43, 95% CI 1.35–1.50) (Fig. 7A, B). None of the included studies differentiated risks associated with indapamide by melanoma subtypes, cumulative doses, or geographic regions.

### Sensitivity analysis

After the inclusions of one duplicated case–control study on hydrochlorothiazide and indapamide in Denmark [42] and one duplicated cohort study in Korea [48], the results were consistent with those of the main analyses (Additional file 1: Table S8). In addition, the sensitivity analysis including only low risk-of-bias studies showed consistent results with those of the main analysis (Additional file 1: Table S9).

## Discussion

Our study indicated an increased risk for NMSC associated with the use of hydrochlorothiazide but not with bendroflumethiazide or indapamide. Specifically, we found a higher risk for SCC than for BCC associated with thiazide use, consistent with evidence that cumulative UV exposure plays a greater role in the aetiology of SCC than of BCC [35, 36]. The minimum average daily UV exposure level to induce skin cancers among thiazide users is unclear; however, one Icelandic study indicated that relatively low levels of average daily UV exposure were sufficient to cause 1.24-fold and 1.14-fold increases in the risks of SCC in situ and BCC, respectively, following hydrochlorothiazide use [13]. Taken together, healthcare professionals and patients should recognize that more aggressive and appropriate photoprotective behaviours (e.g., use of adequate amounts of broad-spectrum sunscreen with a sun-protection factor of ≥ 30) should be re-emphasized to eliminate the carcinogenicity of thiazides [49]. The risk-benefit evaluation in prescribing thiazides must be individually assessed.

The inconsistent risk profiles for skin cancers among individual drugs within the same chemical class of thiazides are possibly due to varying photosensitizing effects under different molar concentrations of the drugs and differences in wavelengths responsible for various histologic types of skin cancers [7]. For example, we found associations of NMSC only with hydrochlorothiazide use, but not with bendroflumethiazide use, possibly due to the shorter half-life of bendroflumethiazide, given the lower molar concentrations of therapeutically equivalent doses [50, 51]. Indapamide has more absorbance in the UV-B spectrum than hydrochlorothiazide in the UV-A spectrum, which plays an essential role in NMSC carcinogenesis [52–54]. In addition, our study suggests that cumulative doses of hydrochlorothiazide under 25,000 mg are relatively safe with regard to BCC, SCC, and melanoma, based on case-control studies. These data could provide an important reference for the selection and dosing of appropriate thiazide drugs taking into consideration the risks for skin cancers.

A similar biological mechanism to NMSC may extend the carcinogenic effects of hydrochlorothiazide to the development of various melanoma subtypes associated with high sun exposure [6, 55]. Previous studies have indicated that different ethnicities have varying risk profiles for melanoma; the risk typically being higher in Caucasians (incidence: 7.6–18.9 per 10,000 people) than in Asians (incidence: 0.5–1.5 per 10,000 people) [56]. Our subgroup analysis on geographic regions revealed reduced melanoma risk associated with hydrochlorothiazide in one case-control study in Taiwan [11], compared with increased risks in five case-control studies in Europe and Australia. Although the dosage of hydrochlorothiazide prescribed in Taiwan is generally lower than that in other non-Asian countries [11], the dose-response analysis found no increased risk of melanoma, especially with medium and high cumulative doses of hydrochlorothiazide, in Taiwan. As thiazide diuretics are not metabolized in the human body [57], possible genetic polymorphisms are unlikely to explain risk differences across populations. Skin photosensitivity reactions are considered important in hydrochlorothiazide carcinogenicity [5]; thus, contradicting results noted between Asian and non-Asian countries may be explained by ethnic differences. For example, ultraviolet exposure plays an important role in the carcinogenesis of melanoma in Caucasians, but not in Asians [56], which is supported by a previous study indicating that acral melanoma, which usually occurs in areas with little to no sun exposure, accounted for 50%–58% of melanoma in Asians [58]. Since a previous study suggested trauma and physical pressure as a risk factor for acral melanoma, the mechanisms of melanoma may vary between Asian and non-Asian populations [59]. Similarly, our subgroup analysis found oppositely directed risks of hydrochlorothiazide-associated NMSC between Asian and non-Asian countries, which may be explained by ethnic differences in skin phototypes. Asian populations typically have larger amounts of melanin (Fitzpatrick skin phototype III or IV), and DNA repair mechanisms are probably more efficient than in fair-skinned Northern European populations [11, 60]. Considering ethnicity as an effect modifier, our findings suggest that hydrochlorothiazide use does not appear to pose a clinically meaningful risk for skin cancers in Asians.

Earlier systematic reviews have reported that thiazides are associated with an increased risk of skin cancers [5, 8, 9]. In comparison with previous reviews, we included more recent studies, with 13 published from February 2019 to January 2022 [10–14, 36–40, 43, 45, 46]. More importantly, our analyses covered a broader range of recent nationwide data, including data from the Brazil, Netherlands, USA, UK, Iceland, Spain, Australia, Taiwan, and Korea. Consequently, our findings could be geographically more generalizable. Our meta-analysis is also the first to evaluate skin cancer risk associated with individual thiazides, instead of pooling all thiazides into one pharmacologic entity. For those with a potential risk of skin cancers, physicians may consider prescribing alternative thiazides such as bendroflumethiazide and indapamide. Furthermore, we conducted subgroup analyses on geographic regions on hydrochlorothiazide and skin cancers. A higher risk for hydrochlorothiazide-associated skin cancers was observed in non-Asian countries than in Asian countries; however, further studies are still needed to understand the aetiology and develop different preventative strategies for Asian skin cancers. Our findings for the different geographic regions support the biological mechanism whereby more sun exposure combined with photosensitizing drugs leads to increased skin cancer risk [61]. The causal link between hydrochlorothiazide and skin cancer was further strengthened by our subgroup analysis on cumulative doses. Notably, no dose-response effect was found between bendroflumethiazide and indapamide use and NMSC, suggesting that skin cancer risk profiles vary with different individual thiazides.

This study has several limitations. First, since we found no eligible RCTs, our findings may be biased through unmeasured confounding, such as lifestyle, diet, amounts of sun exposure, and sun-protective behaviours. Second, high heterogeneity among the studies for NMSC and melanoma was observed in the main analysis, probably due to varying thiazide prescribing patterns and skin cancer incidences across different countries. To identify the sources of heterogeneity across the studies, we performed multiple subgroup analyses to evaluate skin cancer risk, based on different regions and cumulative doses. However, the subgroup analysis did not fully diminish the statistical heterogeneity among the included studies. Third, some results from the meta-analysis were inconsistent between case-control studies and cohort studies on the same outcomes, probably because shorter follow-up durations in the cohort studies may have led to underestimation of the risk estimates [47]. Fourth, the skin cancer risk associated with the use of chlorothiazide, methyclothiazide, and metolazone is unclear due to the lack of relevant data.

## Conclusions

Current evidence supports an increased risk for SCC and melanoma among patients receiving hydrochlorothiazide, but no significant or clinically meaningful risk for those receiving bendroflumethiazide or indapamide. Dermatology consultation and optimal photo-protection may be considered for hydrochlorothiazide users.

## Supplementary Information

**Additional file 1: Supplementary Material. Figure S1**. Forest Plot for the Subgroup Analysis of the Association between Cumulative Doses of Hydrochlorothiazide and Basal Cell Carcinoma in Case-Control Studies. **Figure S2**. Forest Plot for the Subgroup Analysis of the Association between Cumulative Doses of Hydrochlorothiazide and Basal Cell Carcinoma in Cohort Studies. **Figure S3**. Forest Plot for the Subgroup Analysis of the Association between Cumulative Doses of Hydrochlorothiazide and Squamous Cell Carcinoma in Case-Control Studies. **Figure S4**. Forest Plot for the Subgroup Analysis of the Association between Cumulative Doses of Hydrochlorothiazide and Squamous Cell Carcinoma in Cohort Studies**. Figure S5**. Forest Plot for the Subgroup Analysis of the Association between Cumulative Doses of Hydrochlorothiazide and Merkel Cell Carcinoma in Case-Control Studies. **Figure S6**. Forest Plot for the Subgroup Analysis of the Association between Cumulative Doses of Hydrochlorothiazide and Unspecified Non-melanoma Skin Cancer in Cohort Studies. **Figure S7**. Forest Plot for the Subgroup Analysis According to Geographic Regions of Nonmelanoma Skin Cancer in Case-Control Studies of Hydrochlorothiazide (A) Non-Asian countries (B) Asian countries. **Figure S8**. Forest Plot for the Subgroup Analysis According to Geographic Regions of Nonmelanoma Skin Cancer in Cohort Studies of Hydrochlorothiazide (A) Non-Asian countries (B) Asian countries. **Figure S9**. Forest Plot for the Subgroup Analysis of the Association between Cumulative Doses of Hydrochlorothiazide and Melanoma in Case-Control studies. **Figure S10**. Forest Plot for the Subgroup Analysis of the Association between Cumulative Doses of Hydrochlorothiazide and Melanoma in Cohort studies. **Figure S11**. Forest Plot for the Subgroup Analysis According to Geographic Regions of Melanoma in Case-Control Studies of Hydrochlorothiazide. **Figure S12**. Forest Plot for the Subgroup Analysis According to Geographic Regions of Melanoma in Cohort Studies of Hydrochlorothiazide. **Figure S13**. Forest Plot for the Subgroup Analysis According to Melanoma Subtypes in Case-Control Studies of Hydrochlorothiazide. **Figure S14**. Forest Plot for the Subgroup Analysis of the Association between Cumulative Doses of Bendroflumethiazide and Basal Cell Carcinoma in Case-Control Studies. **Figure S15**. Forest Plot for the Subgroup Analysis of the Association between Cumulative Doses of Bendroflumethiazide and Squamous Cell Carcinoma in Case-Control Studies. **Figure S16**. Forest Plot for the Subgroup Analysis of the Association between Cumulative Doses of Bendroflumethiazide and Merkel Cell Carcinoma in Case-Control Studies. **Figure S17**. Forest Plot for the Subgroup Analysis of the Association between Cumulative Doses of Indapamide and Basal Cell Carcinoma in Case-Control Studies. **Figure S18**. Forest Plot for the Subgroup Analysis of the Association between Cumulative Doses of Indapamide and Squamous Cell Carcinoma in Case-Control Studies. **Figure S19**. Forest Plot for the Subgroup Analysis of the Association between Cumulative Doses of Indapamide and Melanoma in Case-Control Studies. **Table S1**. Search Strategy. **Table S2**. Studies with Overlapping Populations. **Table S3**. Exposures for Thiazide Use in the Included Studies. **Table S4**. Other Characteristics of Included Studies. **Table S5**. The Relationship Between Cumulative Duration of Individual Thiazide Uses and Skin Cancer Risk. **Table S6**. Risk-of-bias Assessment of Included Case-Control Studies Based on Newcastle Ottawa Quality Assessment Scale. **Table S7**. Risk-of-bias Assessment of Included Cohort Studies Based on Newcastle Ottawa Quality Assessment Scale. **Table S8**. Comparisons of the Results between the Main and Sensitivity Analyses. **Table S9**. Sensitivity Analysis By Including Only Low Risk-of-bias Case-Control Studies.

## Data Availability

The datasets used and/or analysed during the current study are available from the corresponding author on reasonable request.

## Abbreviations

BCC: Basal cell carcinomas
CI: Confidence interval
HR: Hazard ratio
MCC: Merkel cell carcinoma
MOOSE: Meta-analysis of Observational Studies in Epidemiology guidelines
NMSC: Nonmelanoma skin cancer
NOS: Newcastle-Ottawa Scale
OR: Odds ratio
PRISMA: Preferred Reporting Items for Systematic Reviews and Meta-analyses
RCTs: Randomized controlled trials
RoB: Risk of bias
RR: Risk ratio
SCC: Squamous cell carcinoma
