# Supplementary material for: Associations of thiazide use with skin cancers: a systematic review and meta-analysis
Source: BMC Med. 2022 Jul 7;20:228. doi: 10.1186/s12916-022-02419-9 (PMC9260996; doi:10.1186/s12916-022-02419-9)
Supplement: Supplementary file 1 — Additional file 1: Supplementary Material. Figure S1. Forest Plot for the Subgroup Analysis of the Association between Cumulative Doses of Hydrochlorothiazide and Basal Cell Carcinoma in Case-Control Studies. Figure S2. Forest Plot for the Subgroup Analysis of the Association between Cumulative Doses of Hydrochlorothiazide and Basal Cell Carcinoma in Cohort Studies. Figure S3. Forest Plot for the Subgroup Analysis of the Association between Cumulative Doses of Hydrochlorothiazide and Squamous Cell Carcinoma in Case-Control Studies. Figure S4. Forest Plot for the Subgroup Analysis of the Association between Cumulative Doses of Hydrochlorothiazide and Squamous Cell Carcinoma in Cohort Studies. Figure S5. Forest Plot for the Subgroup Analysis of the Association between Cumulative Doses of Hydrochlorothiazide and Merkel Cell Carcinoma in Case-Control Studies. Figure S6. Forest Plot for the Subgroup Analysis of the Association between Cumulative Doses of Hydrochlorothiazide and Unspecified Non-melanoma Skin Cancer in Cohort Studies. Figure S7. Forest Plot for the Subgroup Analysis According to Geographic Regions of Nonmelanoma Skin Cancer in Case-Control Studies of Hydrochlorothiazide (A) Non-Asian countries (B) Asian countries. Figure S8. Forest Plot for the Subgroup Analysis According to Geographic Regions of Nonmelanoma Skin Cancer in Cohort Studies of Hydrochlorothiazide (A) Non-Asian countries (B) Asian countries. Figure S9. Forest Plot for the Subgroup Analysis of the Association between Cumulative Doses of Hydrochlorothiazide and Melanoma in Case-Control studies. Figure S10. Forest Plot for the Subgroup Analysis of the Association between Cumulative Doses of Hydrochlorothiazide and Melanoma in Cohort studies. Figure S11. Forest Plot for the Subgroup Analysis According to Geographic Regions of Melanoma in Case-Control Studies of Hydrochlorothiazide. Figure S12. Forest Plot for the Subgroup Analysis According to Geographic Regions of Melanoma in Cohort Studies of Hy [file 12916_2022_2419_MOESM1_ESM.docx]

**Additional file 1: Supplementary Material**

**Figure S1.** Forest Plot for the Subgroup Analysis of the Association between Cumulative Doses of Hydrochlorothiazide and Basal Cell Carcinoma in Case-Control Studies

**Figure S2.** Forest Plot for the Subgroup Analysis of the Association between Cumulative Doses of Hydrochlorothiazide and Basal Cell Carcinoma in Cohort Studies

**Figure S3.** Forest Plot for the Subgroup Analysis of the Association between Cumulative Doses of Hydrochlorothiazide and Squamous Cell Carcinoma in Case-Control Studies

**Figure S4.** Forest Plot for the Subgroup Analysis of the Association between Cumulative Doses of Hydrochlorothiazide and Squamous Cell Carcinoma in Cohort Studies

**Figure S5.** Forest Plot for the Subgroup Analysis of the Association between Cumulative Doses of Hydrochlorothiazide and Merkel Cell Carcinoma in Case-Control Studies

**Figure S6.** Forest Plot for the Subgroup Analysis of the Association between Cumulative Doses of Hydrochlorothiazide and Unspecified Nonmelanoma Skin Cancer in Cohort Studies

**Figure S7**. Forest Plot for the Subgroup Analysis According to Geographic Regions of Nonmelanoma Skin Cancer in Case-Control Studies of Hydrochlorothiazide (A) Non-Asian countries (B) Asian countries

**Figure S8**. Forest Plot for the Subgroup Analysis According to Geographic Regions of Nonmelanoma Skin Cancer in Cohort Studies of Hydrochlorothiazide (A) Non-Asian countries (B) Asian countries

**Figure S9.** Forest Plot for the Subgroup Analysis of the Association between Cumulative Doses of Hydrochlorothiazide and Melanoma in Case-control Studies

**Figure S10.** Forest Plot for the Subgroup Analysis of the Association between Cumulative Doses of Hydrochlorothiazide and Melanoma in Cohort Studies

**Figure S11.** Forest Plot for the Subgroup Analysis According to Geographic Regions of Melanoma in Case-Control Studies of Hydrochlorothiazide

**Figure S12.** Forest Plot for the Subgroup Analysis According to Geographic Regions of Melanoma in Cohort Studies of Hydrochlorothiazide

**Figure S13.** Forest Plot for the Subgroup Analysis According to Melanoma Subtypes in Case-Control Studies of Hydrochlorothiazide

**Figure S14.** Forest Plot for the Subgroup Analysis of the Association between Cumulative Doses of Bendroflumethiazide and Basal Cell Carcinoma in Case-Control Studies

**Figure S15.** Forest Plot for the Subgroup Analysis of the Association between Cumulative Doses of Bendroflumethiazide and Squamous Cell Carcinoma in Case-Control Studies

**Figure S16.** Forest Plot for the Subgroup Analysis of the Association between Cumulative Doses of Bendroflumethiazide and Merkel Cell Carcinoma in Case-Control Studies

**Figure S17.** Forest Plot for the Subgroup Analysis of the Association between Cumulative Doses of Indapamide and Basal Cell Carcinoma in Case-Control Studies

**Figure S18.** Forest Plot for the Subgroup Analysis of the Association between Cumulative Doses of Indapamide and Squamous Cell Carcinoma in Case-Control Studies

**Figure S19.** Forest Plot for the Subgroup Analysis of the Association between Cumulative Doses of Indapamide and Melanoma in Case-Control Studies

**Table S1.** Search Strategy.

**Table S2.** Studies with Overlapping Populations

**Table S3.** Exposures for Thiazide Use in the Included Studies

**Table S4.** Other Characteristics of Included Studies

**Table S5.** The Relationship Between Cumulative Duration of Individual Thiazide Use and Skin Cancer Risk

**Table S6.** Risk-of-bias Assessment of Included Case-Control Studies Based on Newcastle Ottawa Quality Assessment Scale

**Table S7.** Risk-of-bias Assessment of Included Cohort Studies Based on Newcastle Ottawa Quality Assessment Scale

**Table S8.** Comparisons of the Results between the Main and Sensitivity Analyses

**Table S9.** Sensitivity Analysis By Including Only Low Risk-of-bias Case-Control Studies

**Figure S1. Forest Plot for the Subgroup Analysis of the Association between Cumulative Doses of Hydrochlorothiazide and Basal Cell Carcinoma in Case-Control Studies.** Low cumulative dose: < 25,000 mg, medium cumulative dose: 25,000-50,000 mg, and high cumulative dose: > 50,000 mg.

**
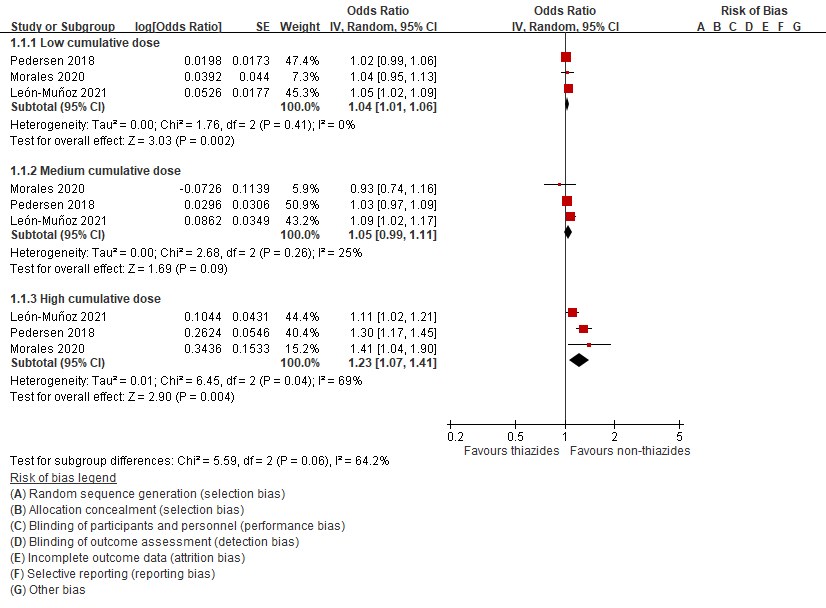
**

**Figure S2. Forest Plot for the Subgroup Analysis of the Association between Cumulative Doses of Hydrochlorothiazide and Basal Cell Carcinoma in Cohort Studies.** Low cumulative dose: < 10,000 mg, medium cumulative dose: 10,000-50,000 mg, and high cumulative dose: > 50,000 mg.


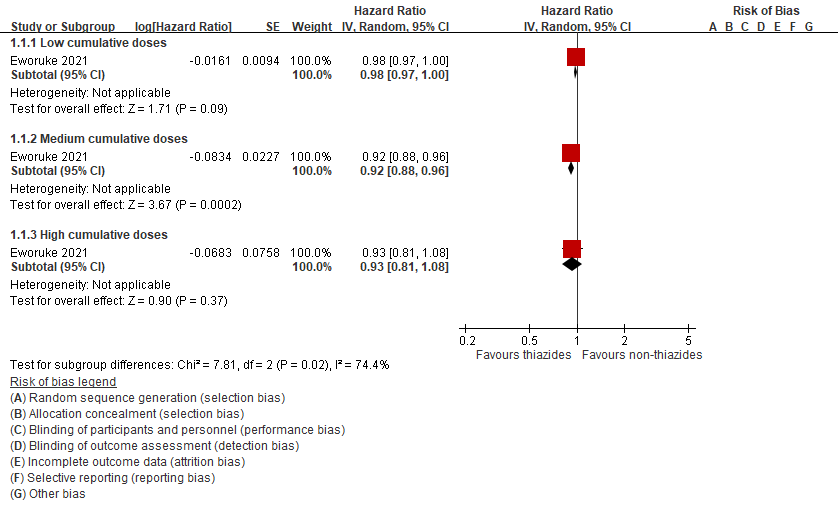


**Figure S3. Forest Plot for the Subgroup Analysis of the Association between Cumulative Doses of Hydrochlorothiazide and Squamous Cell Carcinoma in Case-Control Studies.** Low cumulative dose: < 25,000 mg, medium cumulative dose: 25,000-50,000 mg, and high cumulative dose: > 50,000 mg.


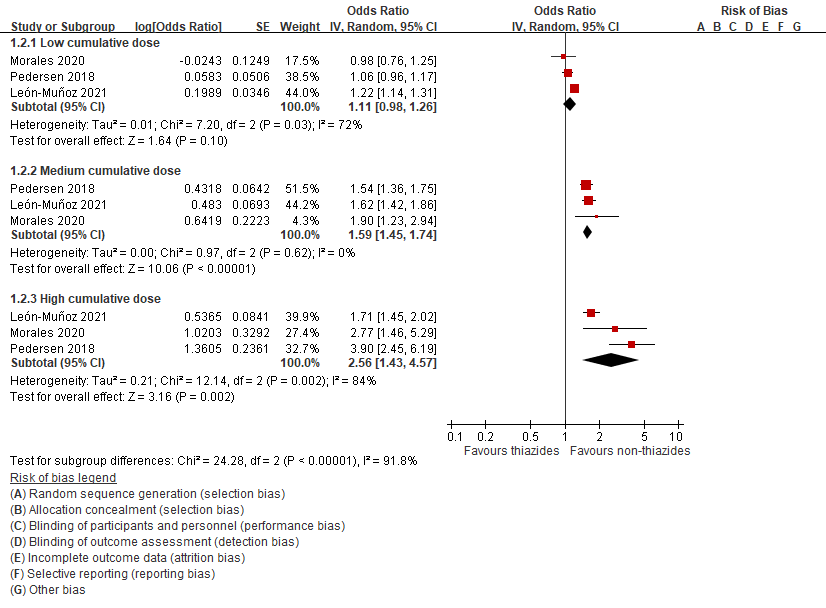


**Figure S4. Forest Plot for the Subgroup Analysis of the Association between Cumulative Doses of Hydrochlorothiazide and Squamous Cell Carcinoma in Cohort Studies.** Low cumulative dose: < 10,000 mg, medium cumulative dose: 10,000-50,000 mg, and high cumulative dose: > 50,000 mg.


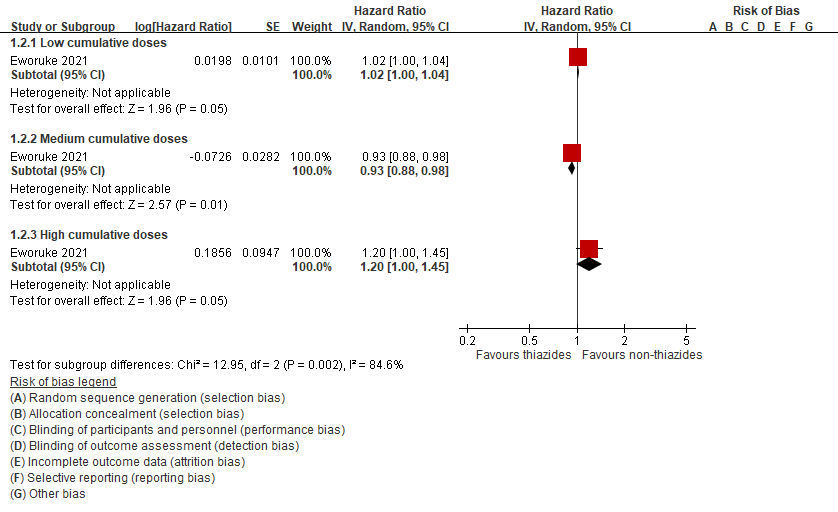


**Figure S5.** **Forest Plot for the Subgroup Analysis of the Association between Cumulative Doses of Hydrochlorothiazide and Merkel Cell Carcinoma in Case-Control Studies.** Low cumulative dose: < 49,999 mg, medium cumulative dose: 50,000-99,999 mg (no data) and high cumulative dose: > 100,000 mg.


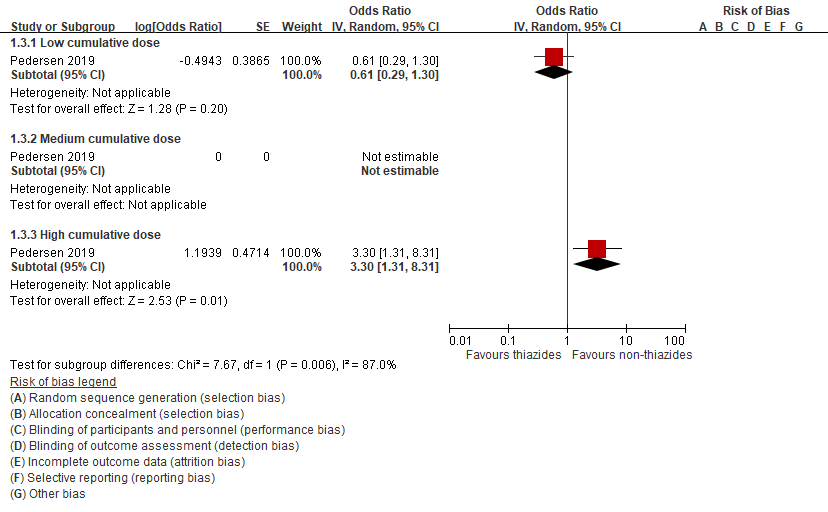


**Figure S6.** **Forest Plot for the Subgroup Analysis of the Association between Cumulative Doses of Hydrochlorothiazide and Unspecified Non-melanoma Skin Cancer in Cohort Studies.** Low cumulative dose: 0-750 mg (SNUH and SNUBH) or 0-250 mg (ASAN), medium cumulative dose: 750-4900 mg (SNUH), 750-5362.5 mg (SNUBH) or 250-1750 mg (ASAN) and high cumulative dose: >4900 mg (SNUH), >5362.5 mg (SNUBH) or >1750 mg (ASAN).


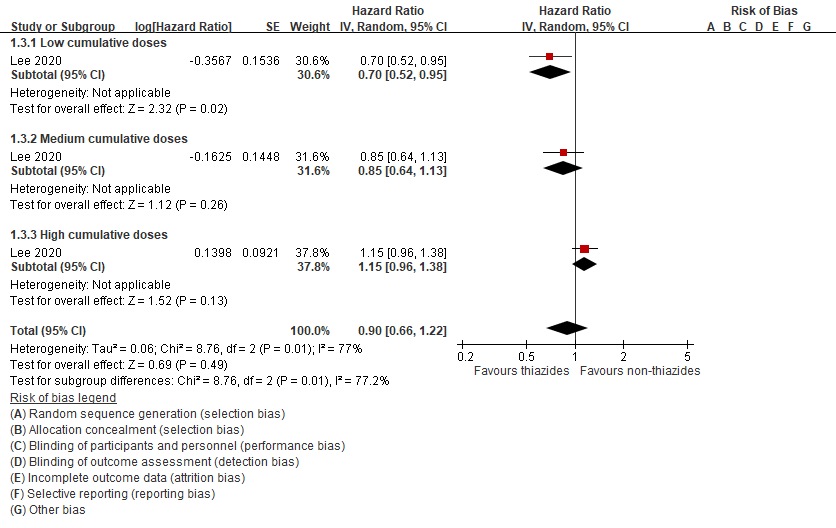


**Figure S7. Forest Plot for the Subgroup Analysis According to Geographic Regions of Non-melanoma Skin Cancer in Case-Control Studies of Hydrochlorothiazide**

**(A) Non-Asian countries**

**
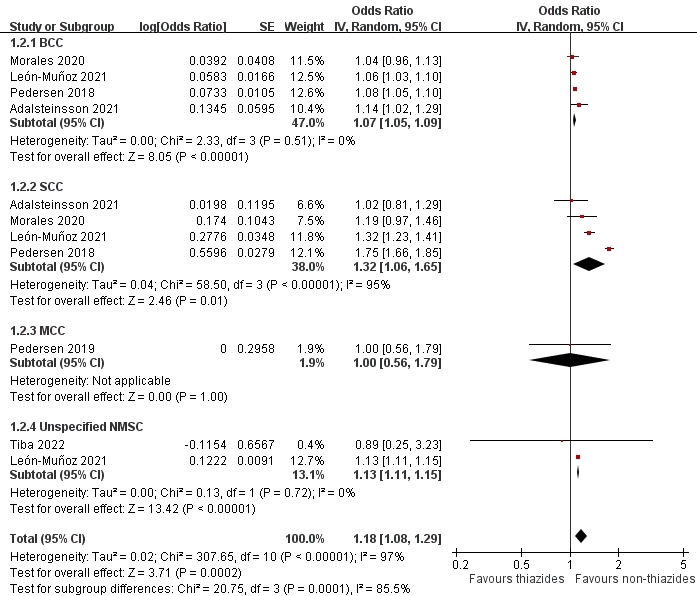
**

**Figure S7. Forest Plot for the Subgroup Analysis According to Geographic Regions of Non-melanoma Skin Cancer in Case-Control Studies of Hydrochlorothiazide**

**(B) Asian countries**

**
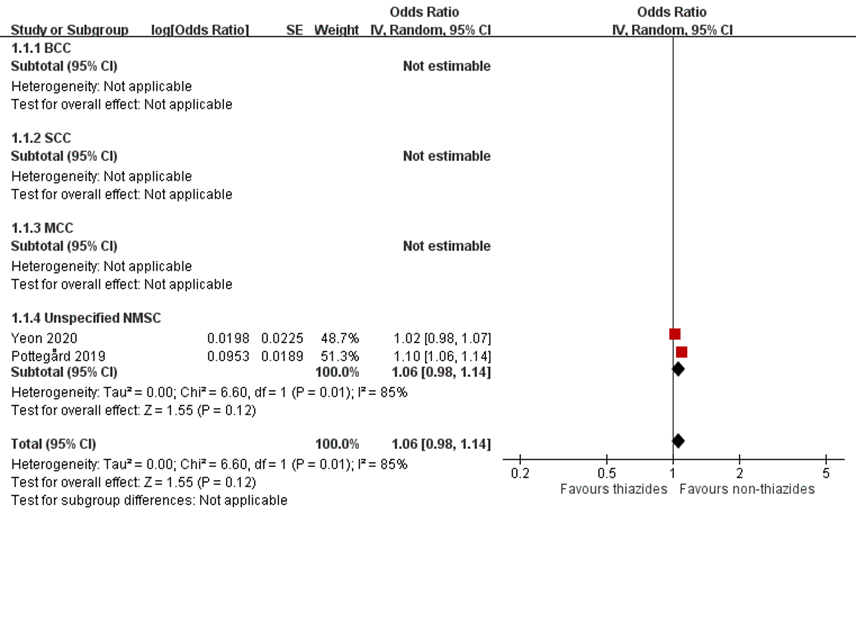
**

**Figure S8. Forest Plot for the Subgroup Analysis According to Geographic Regions of Non-melanoma Skin Cancer in Cohort Studies of Hydrochlorothiazide**

**(A) Non-Asian countries**

**
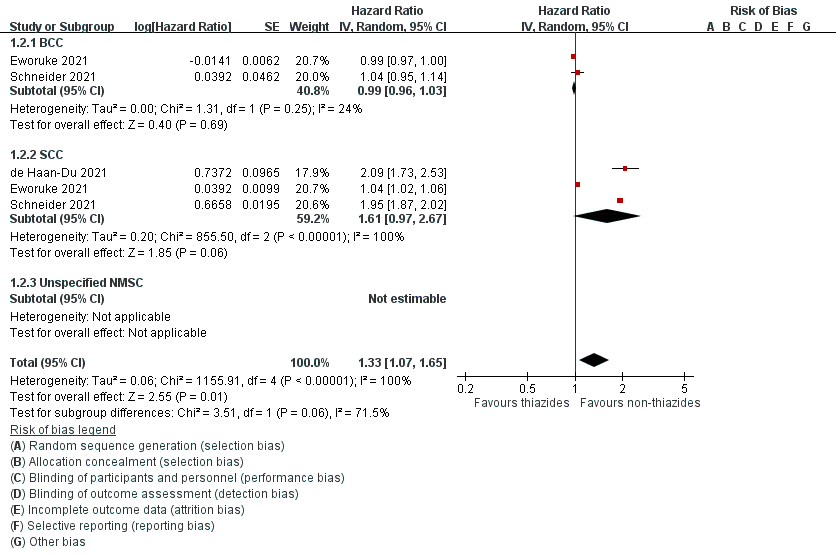
**

**Figure S8. Forest Plot for the Subgroup Analysis According to Geographic Regions of Non-melanoma Skin Cancer in Cohort Studies of Hydrochlorothiazide**

**(B) Asian countries**

**
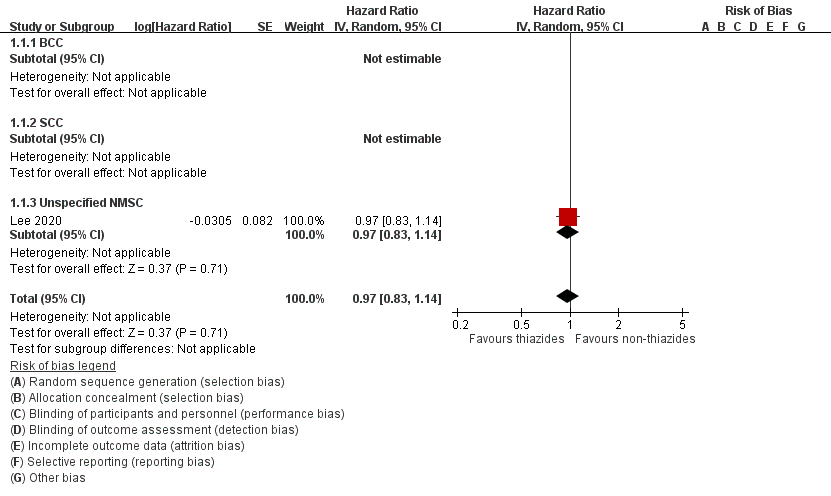
**

**Figure S9.** **Forest Plot for the Subgroup Analysis of the Association between Cumulative Doses of Hydrochlorothiazide and Melanoma in Case-Control studies.** Low cumulative dose: < 25,000 mg, medium cumulative dose: 25,000-50,000 mg, and high cumulative dose: > 50,000 mg.


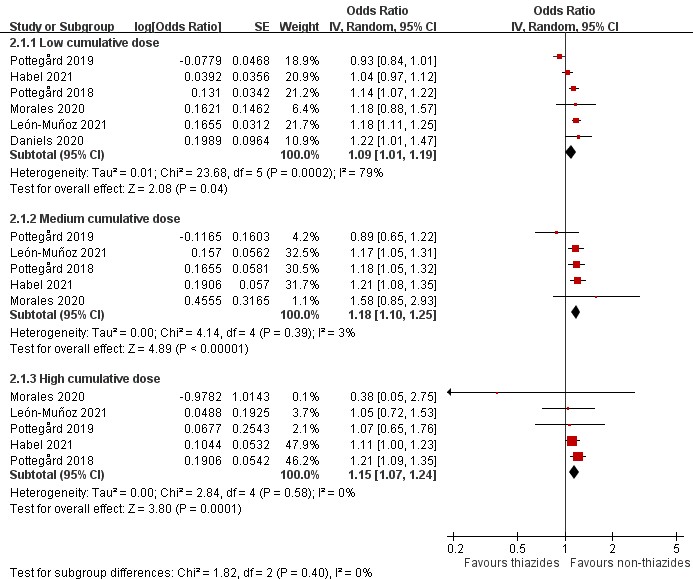


**Figure S10.** **Forest Plot for the Subgroup Analysis of the Association between Cumulative Doses of Hydrochlorothiazide and Melanoma in Cohort studies.** Low cumulative dose: 0-750 mg (SNUH and SNUBH) or 0-250 mg (ASAN), medium cumulative dose: 750-4900 mg (SNUH), 750-5362.5 mg (SNUBH) or 250-1750 mg (ASAN) and high cumulative dose: >4900 mg (SNUH), >5362.5 mg (SNUBH) or >1750 mg (ASAN).


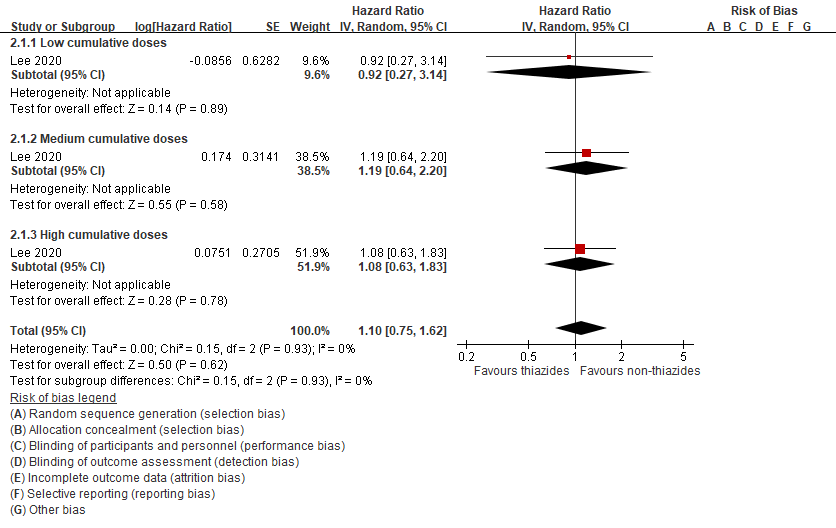


**Figure S11. Forest Plot for the Subgroup Analysis According to Geographic Regions of Melanoma in Case-Control Studies of Hydrochlorothiazide.**

**
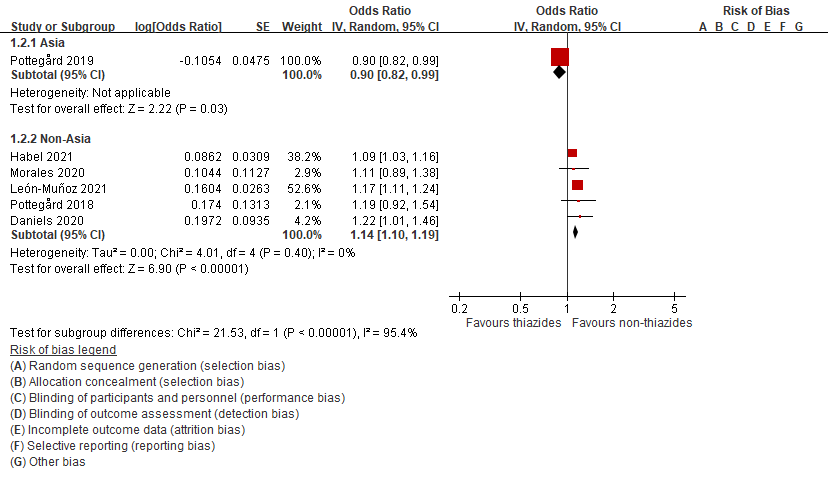
**

**Figure S12.** **Forest Plot for the Subgroup Analysis According to Geographic Regions of Melanoma in Cohort Studies of Hydrochlorothiazide.**


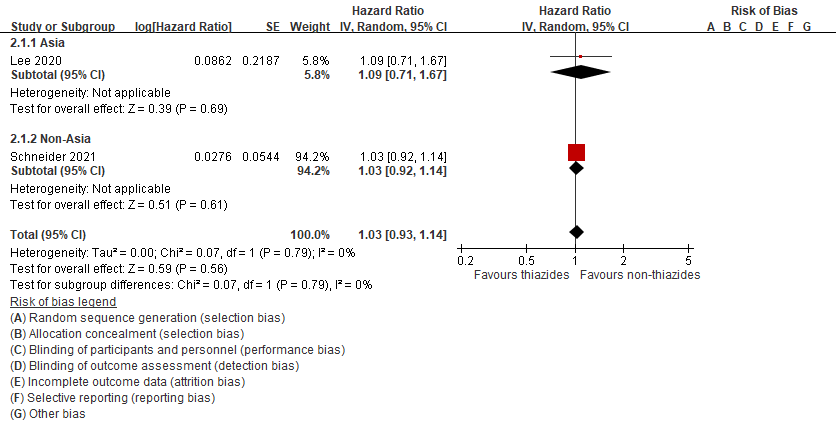


**Figure S13.** **Forest Plot for the Subgroup Analysis According to Melanoma Subtypes in Case-Control Studies of Hydrochlorothiazide.**

**
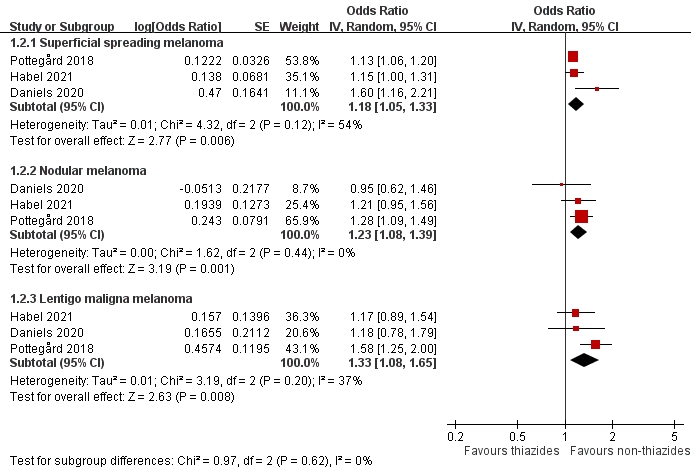
**

**Figure S14. Forest Plot for the Subgroup Analysis of the Association between Cumulative Doses of Bendroflumethiazide and Basal Cell Carcinoma in Case-Control Studies.** Low cumulative dose: < 2,500 mg, medium cumulative dose: 2,500-5,000 mg, and high cumulative dose: > 5,000 mg.

**
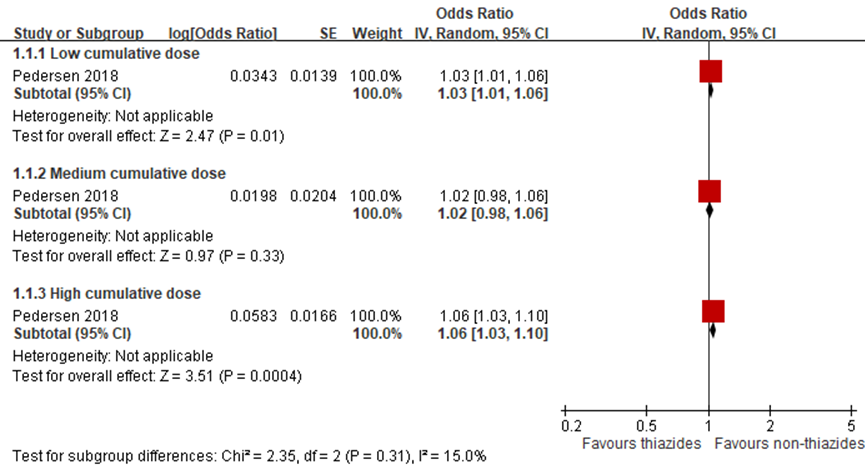
**

**Figure S15. Forest Plot for the Subgroup Analysis of the Association between Cumulative Doses of Bendroflumethiazide and Squamous Cell Carcinoma in Case-Control Studies.** Low cumulative dose: < 2,500 mg, medium cumulative dose: 2,500-5,000 mg, and high cumulative dose: > 5,000 mg.

**
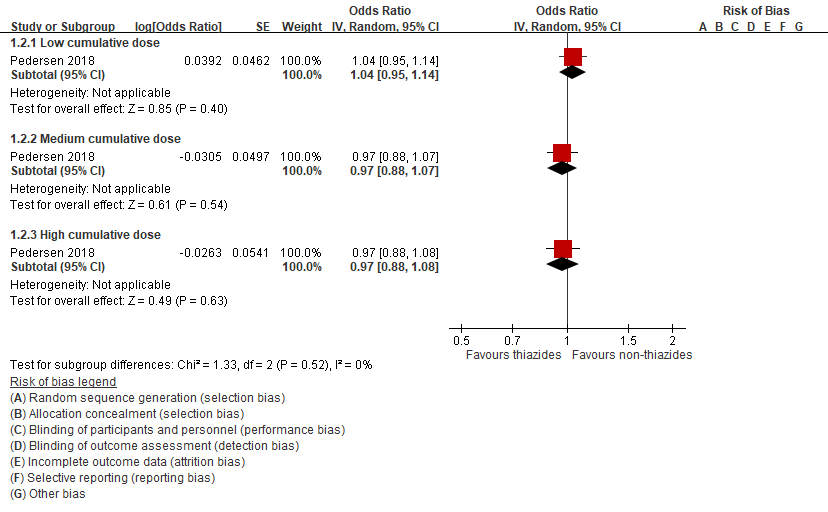
**

**Figure S16. Forest Plot for the Subgroup Analysis of the Association between Cumulative Doses of Bendroflumethiazide and Merkel Cell Carcinoma in Case-Control Studies.** Low cumulative dose: <50,000 mg, medium cumulative dose: 50,000-100,000 mg, and high cumulative dose: > 100,000 mg.


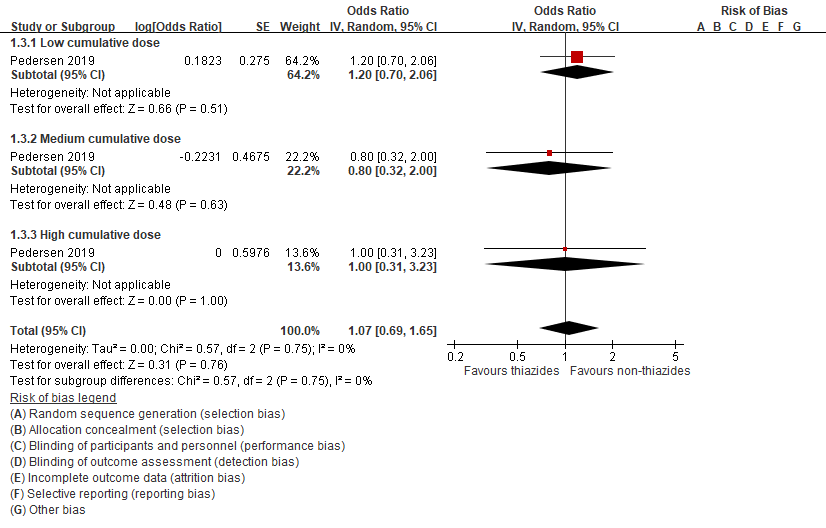


**Figure S17. Forest Plot for the Subgroup Analysis of the Association between Cumulative Doses of Indapamide and Basal Cell Carcinoma in Case-Control Studies.** Low dose: < 1,000 defined daily doses, medium dose: 1,000-2,000 defined daily doses, and high dose: > 2,000 defined daily doses.


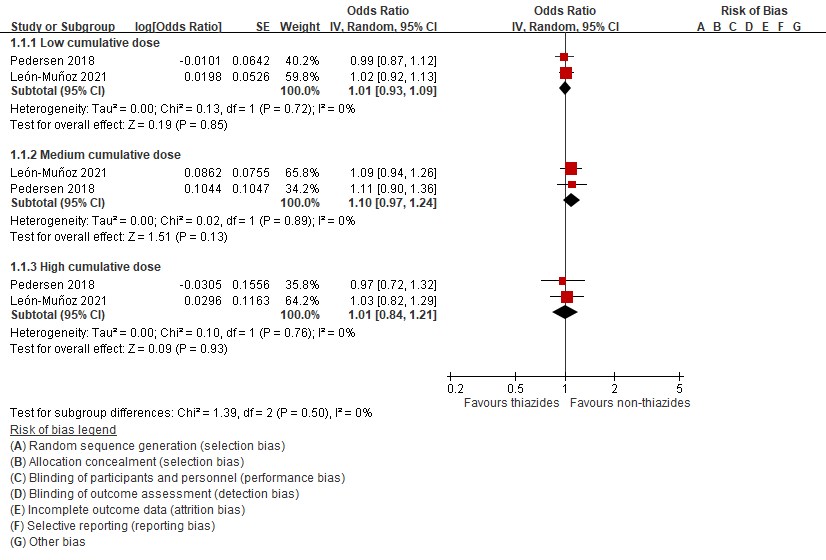


**Figure S18. Forest Plot for the Subgroup Analysis of the Association between Cumulative Doses of Indapamide and Squamous Cell Carcinoma in Case-Control Studies.** Low dose: < 1,000 defined daily doses, medium dose: 1,000-2,000 defined daily doses, and high dose: > 2,000 defined daily doses.

**
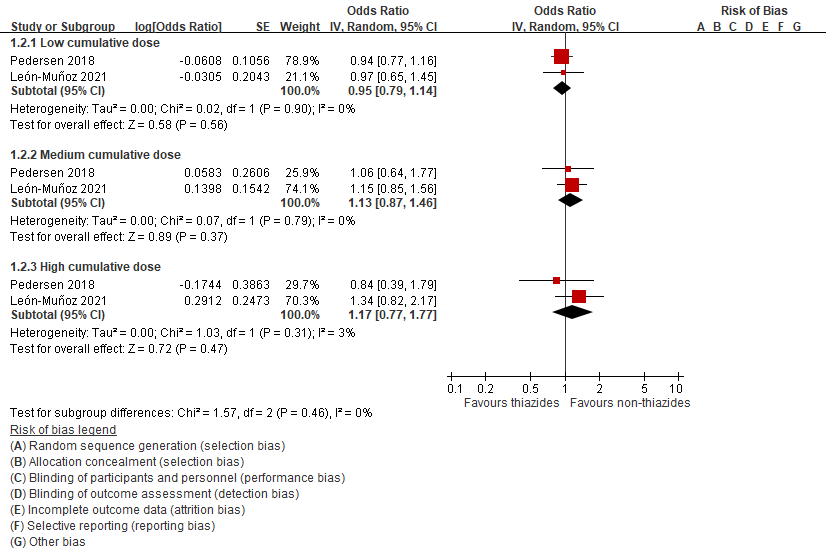
**

**Figure S19. Forest Plot for the Subgroup Analysis of the Association between Cumulative Doses of Indapamide and Melanoma in Case-Control Studies.** Low dose: < 1,000 defined daily doses, medium dose: 1,000-2,000 defined daily doses, and high dose: > 2,000 defined daily doses.
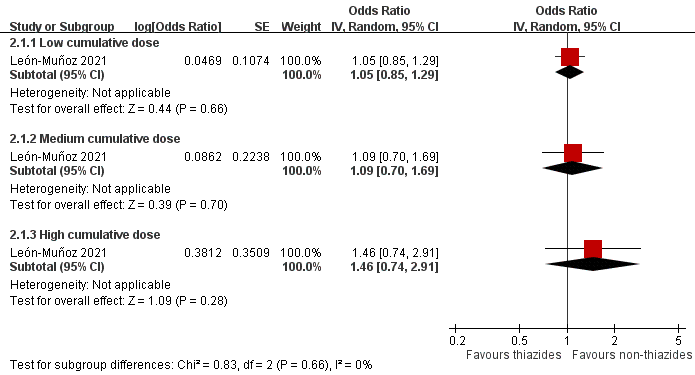


**Table S1.** Search Strategy

| **PubMed** |
| --- |
| 1. "thiazid"[All Fields] OR "thiazides"[MeSH Terms] OR "thiazides"[All Fields] OR "thiazide"[All Fields] OR "thiazidic"[All Fields] OR "thiazide-like"[All Fields] 2. "bendroflumethiazide"[MeSH Terms] OR "bendroflumethiazide"[All Fields] OR "hydroflumethiazide"[MeSH Terms] OR "hydroflumethiazide"[All Fields] OR "hydrochlorothiazid"[All Fields] OR "hydrochlorothiazide"[MeSH Terms] OR "hydrochlorothiazide"[All Fields] OR "chlorothiazide"[MeSH Terms] OR "chlorothiazide"[All Fields] OR "polythiazide"[MeSH Terms] OR "polythiazide"[All Fields] OR "trichlormethiazide"[MeSH Terms] OR "trichlormethiazide"[All Fields] OR "cyclopenthiazide"[MeSH Terms] OR "cyclopenthiazide"[All Fields] OR "methyclothiazide"[MeSH Terms] OR "methyclothiazide"[All Fields] OR "cyclothiazide"[Supplementary Concept] OR "cyclothiazide"[All Fields] OR "mebutizide"[Supplementary Concept] OR "mebutizide"[All Fields] OR "indapamide"[MeSH Terms] OR "indapamide"[All Fields] OR "indapamid"[All Fields] OR "chlorthalidone"[MeSH Terms] OR "chlorthalidone"[All Fields] 3. "skin neoplasms"[MeSH Terms] OR "skin neoplasm"[All Fields] OR "skin neoplasms"[All Fields] OR "skin cancer"[All Fields] OR "skin cancers"[All Fields] 4. "carcinoma, basal cell"[MeSH Terms] OR "basal cell carcinoma"[All Fields] OR "basal cell carcinomas"[All Fields] OR "carcinoma, squamous cell"[MeSH Terms] OR "squamous cell carcinoma"[All Fields] OR "squamous cell carcinomas"[All Fields] OR "merkel cell carcinoma"[All Fields] OR "merkel cell carcinomas"[All Fields] 5. ("melanoma"[MeSH Terms] OR "melanoma"[All Fields] OR "melanomas"[All Fields]) AND ("skin neoplasms"[MeSH Terms] OR "skin neoplasm"[All Fields] OR "skin neoplasms"[All Fields] OR "skin cancer"[All Fields] OR "skin cancers"[All Fields]) 6. ("nonmelanoma"[All Fields] OR "nonmelanomas"[All Fields]) AND ("skin neoplasms"[MeSH Terms] OR "skin neoplasm"[All Fields] OR "skin neoplasms"[All Fields] OR "skin cancer"[All Fields] OR "skin cancers"[All Fields]) 7. (#1 OR #2) AND (#3 OR #4 OR #5 OR #6) |
| **Embase** |
| 1. ('thiazide diuretic agent'/exp OR 'diuretics, thiazide' OR 'diuretics, thiazide and derivatives' OR 'thiazide' OR 'thiazide-like' OR 'thiazide derivative' OR 'thiazide diuretic' OR 'thiazide diuretic agent' OR 'thiazides' OR 'bendroflumethiazide'/exp OR 'hydroflumethiazide'/exp OR 'hydrochlorothiazide'/exp OR 'chlorothiazide'/exp OR 'polythiazide'/exp OR 'trichlormethiazide'/exp OR 'cyclopenthiazide'/exp OR 'methyclothiazide'/exp OR 'cyclothiazide'/exp OR 'mebutizide'/exp OR 'indapamide'/exp OR 'chlorthalidone'/exp) 2. ('skin cancer'/exp OR 'cancer, skin' OR 'malignant skin tumor' OR 'malignant skin tumour' OR 'skin cancer' OR 'skin tumor, malignant' OR 'skin tumour, malignant' OR 'tumor, skin, malignant' OR 'tumour, skin, malignant' OR 'basal cell carcinoma'/exp OR 'basal cell carcinoma' OR 'basal cell epithelioma' OR 'basal cell neoplasms' OR 'basal cell tumor' OR 'basal cell tumour' OR 'basal squamous carcinoma' OR 'basocellular epithelioma' OR 'basosquamous carcinoma' OR 'carcinoma, basal cell' OR 'carcinoma, basosquamous' OR 'epithelioma, basal cell' OR 'neoplasms, basal cell' OR 'skin carcinoma, basal cell type' OR 'basocellular carcinoma' OR 'squamous cell carcinoma'/exp OR 'cancer, squamous cell' OR 'carcinoma, squamous cell' OR 'squamous carcinoma' OR 'squamous cell cancer' OR 'squamous cell carcinoma' OR 'squamous cell epithelioma' OR 'squamous cell neoplasms' OR 'squamous epithelium carcinoma' OR 'neoplasms, squamous cell' OR 'squamous epithelioma' OR 'merkel cell carcinoma'/exp OR 'merkel cell carcinoma' OR (merkel AND ('cell'/exp OR cell) AND ('carcinoma'/exp OR carcinoma)) OR 'melanoma'/exp OR 'malignant melanoma' OR 'malignant melanomatosis' OR 'melanocarcinoma' OR 'melanoma' OR 'melanoma (e)' OR 'melanomalignoma' OR 'non melanoma skin cancer'/exp OR 'non melanoma skin cancer' OR 'nonmelanoma skin cancer') 3. #1 AND #2 |
| **Cochrane Central Register of Controlled Trials** |
| 1. ("thiazide diuretic") OR ("thiazide-like") OR (bendroflumethiazide) OR (hydroflumethiazide) OR (hydrochlorothiazide) OR (chlorothiazide) OR (polythiazide) OR (trichlormethiazide) OR (cyclopenthiazide) OR (methyclothiazide) OR (cyclothiazide) OR (mebutizide) OR (indapamide) OR (chlorthalidone) 2. MeSH descriptor: [Sodium Chloride Symporter Inhibitors] explode all trees 3. "skin cancer" or "basal cell carcinoma" or "squamous cell carcinoma" or "merkel cell carcinoma" or "melanoma" or "nonmelanoma" 4. MeSH descriptor: [Skin Neoplasms] explode all trees 5. (#1 or #2) AND (#3 or #4) |

| **Table S2.** Studies with Overlapping Populations | |
| --- | --- |
| Duplicate studies | Inclusions in the main analyses |
| Case-control studies: hydrochlorothiazide | |
| Denmark:  1. Jensen et al (2008)   1. Data source: Danish Cancer Registry, Civil Registration System 2. Study period: 1989-2003 3. Sample size: 799 patients exposed to hydrochlorothiazide with BCC, SCC or melanoma   2. Pedersen et al (2018)   1. Data source: Danish Cancer Registry, National Prescription Registry, National Patient Registry, Danish Education Registers and Danish Civil Registration System 2. Study period: 2004-2012 3. Sample size: 9,712 patients exposed to hydrochlorothiazide with BCC or SCC   3. Pottegård et al (2018)   1. Data source: Danish Cancer Registry, National Prescription Registry, National Patient Registry, Danish Education Registers and   Danish Civil Registration System   1. Study period: 2004-2015 2. Sample size: 1,958 patients exposed to hydrochlorothiazide with melanoma | Pedersen et al (2018) for BCC and SCC:  Larger sample size (9,712) and more recent time span (2004-2012)  Pottegård et al (2018) for melanoma:  Larger sample size (1,958) and more recent time span (2004-2015) |
| Case-controlled studies: bendroflumethiazide | |
| Denmark:   1. Jensen et al (2008) 2. Data source: Danish Cancer Registry, Civil Registration System 3. Study period: 1989-2003 4. Sample size: 1,310 patients exposed to bendroflumethiazide with BCC or SCC 5. Pedersen et al (2018) 6. Data source: Danish Cancer Registry, National Prescription Registry, National Patient Registry, Danish Education Registers and Danish Civil Registration System 7. Study period: 2004-2012 8. Sample size: 20,665 patients exposed to bendroflumethiazide with BCC or SCC | Pedersen et al (2018) for BCC and SCC  Larger sample size (20,665) and more recent time (2004-2012) |
| Case-control studies: indapamide | |
| Denmark:   1. Jensen et al (2008) 2. Data source: Danish Cancer Registry, Civil Registration System 3. Study period: 1989-2003 4. Sample size: 34 patients exposed to indapamide with BCC or SCC 5. Pedersen et al (2018) 6. Data source: Danish Cancer Registry, National Prescription Registry, National Patient Registry, Danish Education Registers and Danish Civil Registration System 7. Study period: 2004-2012 8. Sample size: 833 patients exposed to indapamide with BCC or SCC | Pedersen et al (2018) for BCC and SCC:  Larger sample size (833) and more recent time (2004-2012) |
| Cohort studies: hydrochlorothiazide | |
| Korea:   1. Kim et al (2021) 2. Data source: Korean National Health Insurance Service 3. Study period: 2002-2013 4. Sample size: 62,243 patients exposed to hydrochlorothiazide with unspecified NMSC 5. Lee et al (2020) 6. Data source: Three Korean hospitals (Seoul National University Hospital, Seoul National University Bundang Hospital, and Asan Medical Center) 7. Study period: 2004-2018 8. Sample size: 149,596 patients exposed to hydrochlorothiazide with unspecified NMSC | Lee et al (2020) for unspecified NMSC:  Larger sample size (149,596) and recent time span (2004-2018) |

| **Table S3.** Exposures for Thiazide Uses in the Included Studies | | | |
| --- | --- | --- | --- |
| Study author | Codes for thiazide exposures | Definition of thiazide exposures | Exposures to combination of thiazides and other anti-hypertensive drugs |
| Case-control study | | | |
| Tiba, 2022 | No mention | ≥1 prescription for hydrochlorothiazide-containing drugs | Yes |
| Adalsteinsson, 2021 | No mention | ≥1 prescription for hydrochlorothiazide-containing drugs | Yes |
| León-Muñoz, 2021 | ATC code:  C03AA03, C07DB01, C09DA01, C09DA02, C09DA03, C09DA04, C09DA06, C09DA07, C09DA08, C09BA01, C09BA02, C09BA03, C09BA05, C09BA06, C09BA07, C09BA08, C09BA09, C09DX01, C09DX03, C07BB02, C07DB01, C09XA52, C09XA54 | ≥1 prescription for hydrochlorothiazide-containing drugs | Yes |
| Habel, 2021 | No mention | ≥1 prescription for hydrochlorothiazide-containing drugs | Yes |
| Morales, 2020 | UK electronic medical records read code:  68680978, 73086978, 76205978, 79881978, 79908979, 81458998, 81459998, 81460998, 81461998, 82196998, 82197998, 83435998  83436998, 83448998, 83449998, 83720998, 83721998, 84176998, 84177998, 84178998, 85218998, 85219998, 86039998, 86040998,  86089998, 86090998, 86092998,  86093998, 86520998, 86521998,  86522998, 87212998, 87213998,  87214998, 87421998, 87422998  87424998, 87426998, 87427998, 87428998, 87515998, 89292997, 89292998, 89831979, 89911979, 90077998, 90433998, 90434998  90547998, 90549998, 91089997, 91089998, 91240998, 91577998, 91679990, 92175990, 92889998, 92890998, 93101998, 93102998  93748997, 93748998, 94185992, 94545998, 94546996, 94549998, 94550998, 94551998, 94633997, 94633998, 94634997, 94634998  94670998, 94671998, 94949990, 94992990, 94993990, 95120992, 95264998, 95267997, 95267998, 95324998, 95853998, 95855998  96209997, 96209998, 96212997, 96212998, 96990998, 97251998, 97551998, 98322979, 98496979, 98499979, 98507979, 98512979  98600998, 99118998, 99151998, 99162997, 99162998, 99266998, 99272998, 99328997, 99328998, 99676998, 99677997, 99677998  99686998, 99819998, 99979979, 99986979 | ≥1 prescription for hydrochlorothiazide-containing drugs | Yes |
| Yeon, 2020 | No mention | ≥1 prescription for hydrochlorothiazide-containing drugs | Yes |
| Daniels, 2020 | ATC code:  C03AA03, C03EA01, C09BA02, C09BA06, C09BA09, C09DA02, C09DA03, C09DA04, C09DA06, C09DA07, C09DA08, C09DX01, C09DX03 | ≥1 prescription for hydrochlorothiazide-containing drugs | Yes |
| Pedersen, 2019 | ATC code:  C03AA03, C03AB, C03EA01, C09DA01, C09DA03, C09DA04, C09DA06,C09DA07, C09DA08, C09BA02, C09BA03, C09BA05, C09DX01, C07BB02, C03AA01, C03AB01 | ≥1 prescriptions of hydrochlorothiazide-containing drugs | Yes |
| Pottegård, 2019 | ATC code:  C03AA03, C03AB, C03EA01, C09DA01, C09DA03, C09DA04, C09DA06, C09DA07, C09DA08, C09BA02, C09BA03, C09BA05, C09DX01, C07BB02 | ≥1 prescriptions of hydrochlorothiazide-containing drugs | Yes |
| Pottegård, 2018 | No mention | ≥1 prescription for hydrochlorothiazide-containing drugs | Yes |
| Pedersen, 2018 | No mention | ≥1 prescription for hydrochlorothiazide-containing drugs | Yes |
| de Vries, 2012 | Not applicable | Self-reported history of a minimum of 3 months of regular intake of thiazide-containing drugs | Yes |
| Jensen, 2008 | ATC code:  C03EA01, C07BB02, C09BA01, C09BA02, C09BA03, C09BA05, C09DA06, C09DA04, C09DA01, C09DA07, C09DA02, C09DA03 C03AA01, C03BA11 | Ever use of hydrochlorothiazide-containing drugs, bendroflumethiazide and indapamide | Unclear |
| Cohort study | | | |
| de Haan-Du J, 2021 | No mention | ≥1 prescription for hydrochlorothiazide-containing drugs | Yes |
| Eworuke, 2021 | No mention | ≥1 prescription for hydrochlorothiazide-containing drugs | Yes |
| Schneider, 2021 | Research group internal system drug code:  B06734, B04569, B04577, B04581, B04590, B04608, B04582, B51052, B06207, B14518, B51002, B52217, B52319, B05770, B06180, B06184, B06196, B06797, B06798, B04556, B04591, B52631, B52307, B06721, B06717, B06750, B04515, B04539, B04544, B04557, B04601, B06146, B06707, B06736, B06741, B06783, B06792, B06731, B06764, B06754, B06758, B06770, B06752, B16703, B05757  B05758, B06150, B04545, B04554, B04618 | ≥1 prescription for hydrochlorothiazide-containing drugs | Yes |
| Lee, 2020 | Korean hospitals (SNUH, SNUBH) drug code:  974226, 974473, 974474, 974642, 974702, 19023453, 19023454, 19078080, 19078101, 19078106, 19102491, 40184184, 40184187, 40184217, 40224166, 40224172, 40224175  Korean hospital (ASANH) drug code:  42925745, 42925750, 42950748,  40044851, 21102246, 21023664, 42950601, 42955595, 43288515, 43293966, 21072870, 21092336, 42950497, 42938852, 42960021, 40165771, 40044817, 40044832, 42938519 | ≥1 prescription for hydrochlorothiazide-containing drugs | Yes |
| Kaae, 2010 | ATC code:  C03AA01, C03AB01 | ≥1 prescription for bendroflumethiazide (bendroflumethiazide and potassium-sparing drugs) | Unclear |

| **Table S4.** Other Characteristics of Included Studies | | | | | |
| --- | --- | --- | --- | --- | --- |
| **First author, Year (Country)** | **Total population** | **Thiazide indication** | **Comorbidities, n (%)**  **(Case or Exposed / Control or Non-exposed)** | | **Adjusted factors** |
|  |  |  | **COPD** | **Diabetes** |  |
| Tiba, 2022 (Brazil) | 89 | Hypertension | NA | NA | Age, sex, smoking, and Fitzpatrick skin phototype |
| Habel, 2021 (US) | 273,957 | Hypertension | NA | NA | Adjusted for age, sex, calendar time, education achieved and socioeconomic level |
| Adalsteinsson, 2021 (Iceland) | 63,381 | Antihypertensive | NA | NA | Photosensitizing medications |
| León-Muñoz, 2021 (Spain) | 1,295,142 | Antihypertensive and diuretic | NA | KC: 20,263 (19) / 195,066 (18.6)  Melanoma: 1,555 (12.1) / 15,409 (12.2) | Age, gender, calendar time, photosensitizing drugs, drugs with suggested antineoplastic effects, use of glucocorticoids, comorbidity |
| Morales, 2020 (United Kingdom) | 2,264,439 | Hypertension, heart failure and oedema | NA | NA | Age, gender, photosensitizing medications, common comorbidities, CCI |
| Yeon, 2020  (Korea) | 10,565 | NA | NA | NA | Age, gender, entry date |
| Pedersen, 2019 (Denmark) | 1,954 | Antihypertensive and diuretic | 10 (10.3) / 144 (7.8) | 19 (19.6) / 187 (10.1) | Age, gender, calendar time, certain drugs, common comorbidities, CCI and highest achieved education |
| Pottegård, 2018  (Denmark) | 212,003 | NA | NA | NA | Age, gender, calendar time, certain drugs, history of skin cancer, common comorbidities, CCI, and highest achieved education |
| Pedersen, 2018 (Denmark) | 1,683,527 | Antihypertensive and diuretic | BCC: 3,093 (4.3) / 66,770 (4.7)  SCC: 642 (7.4) / 10,947 (6.3) | BCC: 3,884 (5.4) / 97,388 (6.8)  SCC: 783 (9.1) / 14,567 (8.4) | Age, gender, calendar time, certain drugs, common comorbidities, CCI and highest achieved education |
| de Vries, 2012  (Several countries) | 2,921 | NA | NA | NA | Age, gender, phototype and country |
| Daniels, 2020  (Australia) | 13,105 | Hypertension | NA | NA | Other diuretics and antihypertensive medicines |
| Pottegård, 2019  (Taiwan) | 357,445 | Antihypertensive | 1,103 (3.8) / 9,585 (3.3) | 5,265 (18.1) / 46,476 (16.0) | Age, gender, calendar time, certain drugs, common comorbidities and CCI |
| Jensen, 2008  (Denmark) | 5,050 | NA | NA | NA | Age, gender and area of residence based on risk set sampling |
| de Haan-Du J, 2021(Netherland) | 71,648 | Antihypertensive | NA | All diabetes patients | Age, gender, diabetes duration,  smoking, systolic blood pressure, body mass index, serum creatinine, baseline year, and comedication |
| Eworuke, 2021  (US) | 10,422,642 | Hypertension and edema | 12.6% | 24.3% | Age, sex, comorbidities, alcohol, comedication |
| Schneider, 2021  (United Kingdom) | 546,417 | Essential hypertension | 7,942 (3.1) / 14,352 (3.8) | 8,577 (3.4) / 14,237 (3.8) | Age, sex, smoking, comorbidities, alcohol, comedication, comorbidities and CCI |
| Lee, 2020  (Korea) | 667,348 | Antihypertensive | SNU: 5,070 (10.7) / 4,747 (10.0)  SNUB: 5,831 (8.9) / 5,583 (8.6)  AMC: 1,459 (4.0) / 1,308 (3.6) | SNU: 17,911 (37.7) / 17,901 (37.6)  SNUB: 21,033 (32.2) / 21,076 (32.3)  AMC: 11,467(31.2) / 11,174 (30.4) | Age, gender, certain drugs, common comorbidities and CCI |
| Kaae, 2010  (Denmark) | NA | NA | NA | NA | Age, period, gender and education |
| *AMC,* Asian Medical Center; *BCC*, Basal cell carcinoma; *CCI*, Charlson Comorbidity Index; *KC,* Keratinocyte carcinoma; *NA*, Not available; *SCC*, Squamous cell carcinoma; *SNU*, Seoul National University Hospital; *SNUB*, Seoul National University Bundang Hospital. | | | | | |

| **Table S5.** The Relationship Between Cumulative Duration of Individual Thiazide Uses and Skin Cancer Risk | | | | | | | |
| --- | --- | --- | --- | --- | --- | --- | --- |
| **Case control study** | | | | | | | |
| **First author (year)** | **Individual thiazide** | **Skin cancer type** | **Ever used** | **1-year prescription** | | **5-years prescription** |  |
| Jensen AØ (2008) | Hydrochlorothiazide | BCC | 1.05 (0.95 – 1.16) | 1.05 (0.94 – 1.17) | | 1.10 (0.95 – 1.26) |  |
|  |  | SCC | 1.58 (1.29 –1.93) | 1.67 (1.36 – 2.07) | | 1.92 (1.46 –2.54) |  |
|  |  | Melanoma | 1.32 (1.03 – 1.70) | 1.30 (0.99 – 1.71) | | 1.24 (0.86 –1.78) |  |
|  | Bendroflumethiazide | BCC | 0.98 (0.90 – 1.06) | 1.00 (0.91 – 1.08) | | 0.98 (0.87 – 1.10) |  |
|  |  | SCC | 1.03 (0.86 –1.22) | 0.91 (0.76 – 1.10) | | 1.03 (0.79 –1.34) |  |
|  |  | Melanoma | 1.08 (0.88 – 1.32) | 1.06 (0.86 – 1.32) | | 1.01 (0.74 –1.37) |  |
|  | Indapamide | BCC | 0.99 (0.63 – 1.56) | 0.94 (0.57 – 1.55) | | 0.90 (0.43 – 1.87) |  |
|  |  | SCC | 1.20 (0.57 –2.54) | 1.10 (0.49 – 2.46) | | 1.02 (0.32 –3.23) |  |
|  |  | Melanoma | 3.30 (1.34 – 8.10) | 3.85 (1.47 – 10.1) | | 6.06 (1.78 –20.7) |  |
| **Cohort studies** | | | | | | | |
| **First author (year)** | **Individual thiazide** | **Skin cancer type** | **< 2 years prescription** | **2-4 years prescription** | | **> 4 years prescription** |  |
| de Haan-Du J (2021) | Hydrochlorothiazide | SCC | 1.18 (1.00-1.40) | 1.57 (1.32-1.88) | | 2.09 (1.73-2.52) |  |
|  | | | **<5.5 years prescription** | | **>5.5 years prescription** | | |
| Schneider R (2021) | Hydrochlorothiazide | BCC | 0.99 (0.94-1.05) | | 1.04 (0.95-1.14) | | |
|  |  | SCC | 1.29 (1.24-1.35) | | 1.95 (1.87-2.02) | | |
|  |  | Melanoma | 1.06 (1.00-1.12) | | 1.03 (0.92-1.14) | | |
|  | Bendroflumethiazide | BCC | 0.95 (0.93-0.97) | | 1.07 (1.04-1.09) | | |
|  |  | SCC | 1.07 (1.05-1.09) | | 1.11 (1.08-1.14) | | |
|  |  | Melanoma | 1.36 (1.23-1.48) | | 1.02 (0.98-1.05) | | |
|  | Indapamide | BCC | 0.98 (0.93-1.03) | | 0.99 (0.91-1.07) | | |
|  |  | SCC | 1.20 (1.15-1.25) | | 0.99 (0.89-1.08) | | |
|  |  | Melanoma | 1.14 (1.08-1.19) | | 1.43 (1.35-1.50) | | |

| **Table S6.** Risk-of-bias Assessment of Included Case-Control Studies Based on Newcastle Ottawa Quality Assessment Scale ^a^ | | | | | | | | | | |
| --- | --- | --- | --- | --- | --- | --- | --- | --- | --- | --- |
| First author (year) | Selection | | | | Comparability | | Exposure | | | Overall RoB^b^ |
|  | Q1 | Q2 | Q3 | Q4 | Q1 | Q2 | Q1 | Q2 | Q3 |  |
| Jensen AØ (2008) | 1 | 1 | 1 | 1 | 1 | 0 | 1 | 1 | 1 | Moderate |
| de Vries E (2012) | 1 | 1 | 0 | 1 | 1 | 1 | 0 | 1 | 0 | High |
| Pedersen SA (2018) | 1 | 1 | 1 | 1 | 1 | 1 | 1 | 1 | 1 | Low |
| Pottegård A (2018) | 1 | 1 | 1 | 1 | 1 | 1 | 0 | 1 | 0 | Moderate |
| Pedersen SA (2019) | 1 | 1 | 1 | 1 | 1 | 1 | 1 | 1 | 1 | Low |
| Pottegård A (2019) | 0 | 1 | 1 | 1 | 1 | 1 | 1 | 1 | 1 | Moderate |
| Morales DR (2020) | 0 | 1 | 1 | 1 | 1 | 1 | 1 | 1 | 1 | Moderate |
| Daniels B (2020) | 1 | 1 | 1 | 1 | 1 | 1 | 1 | 1 | 1 | Low |
| Bora Yeon (2020) | 0 | 1 | 1 | 1 | 1 | 0 | 1 | 1 | 1 | Moderate |
| Adalsteinsson JA (2021) | 1 | 1 | 1 | 1 | 0 | 1 | 1 | 1 | 1 | Moderate |
| Habel LA (2021) | 1 | 1 | 1 | 1 | 1 | 1 | 1 | 1 | 0 | Moderate |
| León-Muñoz LM (2021) | 1 | 1 | 1 | 1 | 1 | 1 | 1 | 1 | 1 | Low |
| Tiba APL (2022) | 0 | 0 | 1 | 1 | 1 | 1 | 0 | 1 | 0 | High |
| RoB, risk of bias.  ^a^The judgments of each item in the selection, comparability and exposure are as following:   1. Selection:   Q1: Is the case definition adequate? 1: Histologically confirmed diagnosis / 0: Record linkage (e.g., the uses of diagnosis codes) or not stated.  Q2: Representativeness of the cases? 1: Consecutive representative series of cases (e.g., time period during 2000-2012) / 0: Potential for selection biases or not stated.  Q3: Selection of controls? 1: Community controls / 0: Hospital controls or not stated.  Q4: Definition of controls? 1: No history of diseases (e.g., cancer-free controls) / 0: Not stated.   1. Comparability:   Q1. Study adjusted for age and gender? 1: yes / 0: no.  Q2. Study adjusted for any medications, race or calendar time? 1: yes / 0: no.   1. Exposure:   Q1. Ascertainment of exposure? 1: Secure record (e.g., Registry data, healthcare system data, health insurance data) / 0: Self-report data, self-report questionnaires or not stated.  Q2. Same method of ascertainment for cases and controls? 1: Yes / 0: No or not stated.  Q3. Non-Response rate? 1: Nationwide data sources / 0: Regional data sources or not stated  ^b^We considered studies with a score of 9 stars to be at low RoB, studies that scored 7 or 8 stars at moderate RoB, and those that scored 6 stars or less at high RoB. | | | | | | | | | | |

| **Table S7.** Risk-of-bias Assessment of Included Cohort Studies Based on Newcastle Ottawa Quality Assessment Scale^a^ | | | | | | | | | | |
| --- | --- | --- | --- | --- | --- | --- | --- | --- | --- | --- |
| First author (year) | Selection | | | | Comparability | | Outcome | | | Overall RoB^b^ |
|  | Q1 | Q2 | Q3 | Q4 | Q1 | Q2 | Q1 | Q2 | Q3 |  |
| Kaae J (2010) | 1 | 0 | 1 | 0 | 1 | 1 | 1 | 0 | 1 | High |
| Lee SM (2012) | 1 | 1 | 1 | 1 | 1 | 1 | 1 | 0 | 0 | Moderate |
| Schneider R (2021) | 1 | 1 | 1 | 1 | 1 | 1 | 1 | 0 | 1 | Moderate |
| Eworuke E (2021) | 1 | 1 | 1 | 1 | 1 | 1 | 1 | 0 | 1 | Moderate |
| de Haan-Du J (2021) | 1 | 1 | 1 | 1 | 1 | 1 | 1 | 0 | 0 | Moderate |
| RoB, risk of bias.  ^a^The judgments of each item in the selection, comparability and exposure are as following:   1. Selection:   Q1. Representativeness of exposed cohort: 1: Truly or somewhat representative of a community / population-based study (e.g., National data sources) / 0: selected group of users or lack of description of the derivation of the cohort  Q2. Selection of non-exposed cohort: 1: Drawn from the same community as the exposed cohort / 0, drawn from a different source or lack of description of the derivation of the non-exposed cohort.  Q3. Ascertainment of exposure: 1: Secure record or structured interview (e.g., Registry data, healthcare system data, health insurance data) / 0: written self-report or lack of description of validation.   1. Comparability:   Q1. Study adjusted for age and gender? 1: yes / 0: no  Q2. Study adjusted for any medications, race, calendar time? 1: yes / 0: no   1. Outcome:   Q1. Assessment of outcome? 1: Independent blind assessment, confirmed by medical records or record linkage (e.g., the uses of diagnosis codes or WHO histological codes) / 0: Self-reported or no description.  Q2. Was follow-up long enough for outcomes to occur? 1: Duration of follow-up at least 10 years / 0: Duration of follow-up < 10 years  Q3. Loss to follow-up rate? 1: Complete follow-up of loss to follow-up rate less than 20% / 0: Loss to follow-up rate more than 20% or no statement.  ^b^We considered studies with a score of 9 stars to be at low RoB, studies that scored 7 or 8 stars at moderate RoB, and those that scored 6 stars or less at high RoB. | | | | | | | | | | |

| **Table S8.** Comparisons of the Results between Main and Sensitivity Analyses | | | | |
| --- | --- | --- | --- | --- |
|  | Main analyses | | Sensitivity Analyses | |
| Case-control studies (+ in sensitivity analyses) | Pooled ORs | 95% CI | Pooled ORs | 95% CI |
| **Hydrochlorothiazide** |  |  |  |  |
| NMSC | 1.16 | (1.08-1.24) | 1.16 | (1.09-1.25) |
| BCC (+ Jensen et al, 2008) | 1.07 | (1.05-1.09) | 1.07 | (1.05-1.09) |
| SCC (+ Jensen et al, 2008) | 1.32 | (1.06-1.65) | 1.37 | (1.13-1.65) |
| MCC | 1.00 | (0.56-1.79) | 1.00 | (0.56-1.79) |
| Unspecific NMSC | 1.09 | (1.03-1.15) | 1.09 | (1.03-1.15) |
| Melanoma (+ Jensen et al, 2008) | 1.11 | (1.02-1.20) | 1.12 | (1.04-1.21) |
|  |  |  |  |  |
| **Bendroflumethiazide** |  |  |  |  |
| NMSC | 1.05 | (0.99-1.12) | 1.03 | (0.98-1.08) |
| BCC (+ Jensen et al, 2008) | 1.07 | (0.91-1.26) | 1.02 | (0.97-1.08) |
| SCC (+ Jensen et al, 2008) | 1.26 | (0.78-2.02) | 1.11 | (0.93-1.34) |
| MCC | 1.13 | (0.70-1.82) | 1.13 | (0.70-1.82) |
|  |  |  |  |  |
| **Indapamide** |  |  |  |  |
| NMSC | 1.01 | (0.96-1.05) | 1.01 | (0.96-1.05) |
| BCC (+ Jensen et al, 2008) | 1.01 | (0.96-1.06) | 1.01 | (0.96-1.06) |
| SCC (+ Jensen et al, 2008) | 0.98 | (0.87-1.09) | 0.98 | (0.88-1.09) |
|  |  |  |  |  |
| Cohort studies (+ in sensitivity analyses) | Pooled HRs | 95% CI | Pooled HRs | 95% CI |
| **Hydrochlorothiazide** |  |  |  |  |
| NMSC | 1.26 | (1.04-1.54) | 1.28 | (1.06-1.55) |
| BCC | 0.99 | (0.96-1.03) | 0.99 | (0.96-1.03) |
| SCC | 1.61 | (0.97-2.67) | 1.61 | (0.97-2.67) |
| Unspecific NMSC (+ Kim et al, 2021) | 0.97 | (0.83-1.14) | 1.16 | (0.77-1.75) |

*BCC*, Basal cell carcinoma; *CI*, Confidence interval; *HR*, hazard ratio; *MCC*, Merkel cell carcinoma; *NMSC*, Nonmelanoma skin cancer; *OR*, Odds ratio; *SCC*, Squamous cell carcinoma

| **Table S9.** Sensitivity Analysis By Including Only Low Risk-of-bias Case-Control Studies^a^ | | |
| --- | --- | --- |
|  | Pooled ORs | 95% CI |
| **Hydrochlorothiazide** |  |  |
| NMSC | 1.24 | (1.11-1.39) |
| BCC | 1.07 | (1.05-1.09) |
| SCC | 1.52 | (1.15-2.01) |
| MCC | NA | NA |
| Unspecific NMSC | 1.13 | (1.11-1.15) |
| Melanoma | 1.18 | (1.12-1.24) |
|  |  |  |
| **Bendroflumethiazide** |  |  |
| NMSC | 1.03 | (1.01-1.05) |
| BCC | 1.03 | (1.01-1.05) |
| SCC | 1.02 | (0.97-1.08) |
| MCC | NA | NA |
| Unspecific NMSC | NA | NA |
| Melanoma | NA | NA |
|  |  |  |
| **Indapamide** |  |  |
| NMSC | 1.01 | (0.96-1.05) |
| BCC | 1.01 | (0.96-1.06) |
| SCC | 0.98 | (0.87-1.09) |
| MCC | NA | NA |
| Unspecific NMSC | NA | NA |
| Melanoma | 1.11 | (0.95-1.30) |

^a^ No included cohort studies were judged as having low risk of bias.

*BCC*, Basal cell carcinoma; *CI*, Confidence interval; *HR*, hazard ratio; *MCC*, Merkel cell carcinoma; *NA*, not available; *NMSC*, Nonmelanoma skin cancer; *OR*, Odds ratio; *SCC*, Squamous cell carcinoma
